# Blue‐light receptor phototropin 1 suppresses immunity to promote *Phytophthora infestans* infection

**DOI:** 10.1111/nph.17929

**Published:** 2022-01-08

**Authors:** Shaista Naqvi, Qin He, Franziska Trusch, Huishan Qiu, Jasmine Pham, Qingguo Sun, John M. Christie, Eleanor M. Gilroy, Paul R. J. Birch

**Affiliations:** ^1^ Division of Plant Sciences James Hutton Institute University of Dundee School of Life Sciences Errol Rd Invergowrie, Dundee DD2 5DA UK; ^2^ Key Laboratory of Horticultural Plant Biology (HZAU) Ministry of Education Key Laboratory of Potato Biology and Biotechnology (HZAU) Ministry of Agriculture and Rural Affairs Huazhong Agricultural University Wuhan Hubei 430070 China; ^3^ Institute of Molecular, Cell and Systems Biology College of Medical, Veterinary, and Life Sciences University of Glasgow Glasgow G12 8QQ UK; ^4^ Cell and Molecular Science James Hutton Institute Invergowrie, Dundee DD2 5DA UK

**Keywords:** disease resistance, effector, late blight, *Phytophthora*, plant immunity, susceptibility

## Abstract

- Blue‐light (BL) phototropin receptors (phot1 and phot2) regulate plant growth by activating NPH3/RPT2‐like (NRL) family members. Little is known about roles for BL and phots in regulating plant immunity. We showed previously that *Phytophthora infestans* RXLR effector Pi02860 targets potato (St)NRL1, promoting its ability to enhance susceptibility by facilitating proteasome‐mediated degradation of the immune regulator StSWAP70. This raises the question: do BL and phots negatively regulate immunity?
- We employed coimmunoprecipitation, virus‐induced gene silencing, transient overexpression and targeted mutation to investigate contributions of phots to regulating immunity.
- Whereas transient overexpression of Stphot1 and Stphot2 enhances *P*. *infestans* colonization of *Nicotiana benthamiana*, silencing endogenous *Nbphot1* or *Nbphot2* reduces infection. Stphot1, but not Stphot2, suppressed the INF1‐triggered cell death (ICD) immune response in a BL‐ and NRL1‐dependent manner. Stphot1, when coexpressed with StNRL1, promotes degradation of StSWAP70, whereas Stphot2 does not. Kinase‐dead Stphot1 fails to suppress ICD, enhance *P. infestans* colonization or promote StSWAP70 degradation. Critically, BL enhances *P. infestans* infection, which probably involves phots but not other BL receptors such as cryptochromes and F‐box proteins ZTL1 and FKF1.
- We demonstrate that Stphot1 and Stphot2 play different roles in promoting susceptibility, and Stphot1 kinase activity is required for BL‐ and StNRL1‐mediated immune suppression.

## Introduction

Light is essential for plant growth and development. Plants sense light intensity, duration, quality and direction using a variety of photoreceptors that detect different wavelengths. These include: UV resistance locus 8 (Rizzini *et al*., 2011) associated with ultraviolet B (UV‐B) perception; phytochromes (Quail *et al*., 1995) associated with red/far red light perception; and cryptochromes (Cashmore *et al*., 1999), phototropins (phot1 and phot2; Briggs *et al*., 2001) and the Kelch containing F‐Box protein (KFB) subfamily (Suetsugu & Wada, 2013) involved in blue light (BL) perception.

Phots belong to the AGC (cAMP‐dependent protein kinase A, cGMP‐dependent protein kinase G, and phospholipid‐dependent protein kinase C) family of kinases with an N‐terminal photosensory region composed of two light oxygen voltage (LOV) domains (LOV1 and LOV2) and a C‐terminal Ser/Thr kinase domain. LOV domains act as molecular switches by regulating the activity of their C‐terminal kinase domain. BL sensing triggers covalent binding of the flavin mononucleotide (FMN) chromophore to each LOV domain, leading to conformational changes in the protein, which results in their autophosphorylation and subsequent phosphorylation of their substrate proteins (Christie *et al*., 1999). Kinase‐inactive phot mutants are nonfunctional, highlighting the importance of receptor autophosphorylation and substrate phosphorylation for signalling (Christie *et al*., 2015). Phots optimize photosynthetic productivity by coordinating multiple light‐capturing processes. These include chloroplast relocation movements (Kong & Wada, 2014), stomatal opening (Inoue & Kinoshita, 2017) and phototropism (Christie & Murphy, 2013; Briggs, 2014), all of which influence photosynthetic competence by improving the efficiency of light capture, reducing photodamage, and regulating gas exchange between leaves and the atmosphere (Christie *et al*., 2015). Two BTB/POZ proteins, NON‐PHOTOTROPIC HYPOCOTYL 3 (NPH3) and ROOT PHOTOTROPISM 2 (RPT2), function as signal transducers downstream of phots (Liscum *et al*., 2014; Christie *et al*., 2018) and are founding members of the NRL family, which contains over 30 members in Arabidopsis (Gingerich *et al*., 2007). NRL proteins play key roles in establishing phot‐mediated responses. NPH3 and RPT2 are involved in auxin‐mediated responses such as phototropism, leaf positioning and flattening (Christie *et al*., 2015), whereas ‘NRL PROTEIN FOR CHLOROPLAST MOVEMENT 1’ (NCH1) acts with RPT2 to mediate chloroplast accumulation movement (Suetsugu *et al*., 2016). While red light and UV‐B perception have been implicated in regulating biotic stress responses in plants (e.g. Ballaré, 2014; Ballaré & Pierek, 2017), little information is available regarding a role for BL perception in regulating plant immunity.

The inducible immune system in plants comprises two levels, pattern‐triggered immunity (PTI) and effector‐triggered immunity (ETI). PTI is activated when pattern recognition receptors (PRRs), at the cell surface, detect conserved plant/microbe‐associated molecular patterns (P/MAMPs) from invading pathogens (Jones & Dangl, 2006). Examples of P/MAMPs include elicitin proteins from oomycetes, such as infestin1 (INF1) from *Phytophthora infestans* (Domazakis *et al*., 2018). Adapted pathogens deliver effector proteins that act either inside or outside of plant cells to suppress PTI. In turn, a second layer of plant immunity can be activated if cytoplasmic nucleotide‐binding leucine‐rich repeat resistance proteins detect the presence of effectors, leading to ETI (Jones & Dangl, 2006).

Oomycetes, such as the devastating potato blight pathogen *P*. *infestans*, deliver effector proteins with a conserved Arg–any amino acid–Leu–Arg (RXLR) motif into plant cells during infection. RXLR effectors manipulate host proteins and processes to cause disease, often by suppressing PTI (Whisson *et al*., 2016; Wang *et al*., 2017; Boevink *et al*., 2020; He *et al*., 2020). Previously, we showed that the *P*. *infestans* RXLR effector Pi02860 targets a potato NRL family member, StNRL1, which acts as a susceptibility (S) factor to promote *P. infestans* colonization, in that it is an endogenous negative regulator of immunity (Yang *et al*., 2016). NRL proteins such as NPH3 and NCH1 interact with Cullin 3A (Cul3A) in Arabidopsis and have been proposed to form Cul3‐based E3 ubiquitin ligase (CRL3) complexes that mediate the ubiquitination of their targets (Roberts *et al*., 2011; Zhang *et al*., 2014). Recently, we reported a substrate for StNRL1, the guanine nucleotide exchange factor StSWAP70, which is required for the INF1‐triggered cell death (ICD) immune response (He *et al*., 2018). StNRL1 targets StSWAP70 and causes its proteasome‐mediated degradation, hence suppressing ICD. The *P. infestans* effector Pi02860 enhanced the interaction between StNRL1 and StSWAP70, promoting turnover of the latter (He *et al*., 2018). A key question arising from these previous studies concerns the nature and components of the endogenous system that negatively regulates immunity. As phots act upstream of NRLs to transduce BL perception into a range of physiological responses (Christie *et al*., 2018), we investigated their potential roles in regulating StNRL1 to suppress immunity and enhance *P. infestans* infection in our model system, *Nicotiana benthamiana*.

## Materials and Methods

### Plant material and growth conditions

*Nicotiana benthamiana* plants were grown under a cycle of 16 h day at 22°C and 8 h night at 18°C. Supplementary lighting and shading were automatically provided when ambient light was below 200 W m^−2^ or above 450 W m^−2^, respectively. For monochromatic light experiments, LED Proflex lights (LED Technologies, Cheshire, UK) were used: white (100.622; 395–730 nm, 50 µmol m^−2^ s^−1^), red (100.277; 620–630 nm, 20 µmol m^−2^ s^−1^) and blue (100.272; 450–465 nm, 50 µmol m^−2^ s^−1^).

### Vector construction

Full‐length Stphot1 (PGSC0003DMT400065251/XM_006365087), Stphot2 (XM_006347729), StNPH3 (PGSC0003DMT400083738/XM_006351681) and StRPT2 (PGSC0003DMT400031245/XM_006353666) were synthesized in PUC57 (GenScript, Piscataway, NJ, USA) using sequence information available in databases. Synthesized genes were cloned into pDONR201 (Invitrogen/Thermo Fisher Scientific, Waltham, MA, USA) to generate entry clones via BP reactions using Gateway technology (Invitrogen). Primer sequences are shown in Supporting Information Table S1. Protein fusions were made with N‐terminal GFP, mRFP or cMYC by recombining the entry clones with LR clonase into vectors pB7WGF2, pK7WGR2 and pGWB18, respectively.

### Mutagenesis

The Stphot1 kinase‐dead mutant was generated according to the manufacturer’s protocol described for the QuickChange Site‐Directed Mutagenesis XL II Kit (Agilent, Santa Clara, CA, USA) using pDONR201‐Stphot1 as a template. The primer sequences used for mutation are shown in Table S1. A conserved residue in Stphot1, Asp at position 832 (D832), was mutated to Asn (N) generating Stphot1.KD, which results in a kinase‐dead version of Stphot1. The mutant Stphot1.KD was recombined, using LR clonase (Invitrogen), into pB7WGF2 or pK7WGR2 vectors for *in planta* expression and *in vitro* kinase assays.

### Agrobacterium‐mediated transient expression

Constructs used in this work were transformed into *Agrobacterium tumefaciens* strain AGL1 VirG pSOUP using electroporation. *Agrobacterium* cultures containing constructs were grown in yeast extract beef (YEB) broth with suitable antibiotics and shaking at 28°C overnight. The cultures were spun down at 4500 g to obtain bacterial pellets, which were resuspended in infiltration buffer (10 mM MES, 10 mM MgCl_2_ and 200 mM acetosyringone). Bacterial densities were adjusted to obtain a required OD_600_ before infiltration into *N. benthamiana* leaves (0.5 for immunoblots, immunoprecipitation and cell death assays, 0.15 for *P. infestans* infection assays). To express multiple constructs together, *Agrobacterium* cultures containing various constructs were mixed together before infiltrations. For infection assays, *Agrobacterium* suspensions were infiltrated into leaves and each infiltration site was inoculated with 10 μl of *P. infestans* inoculum at 80 000 sporangia ml^–1^ 24 h after infiltration. Lesion sizes were measured at 7–8 d post‐ inoculation (dpi). For cell death assays, the number of positive hypersensitive response sites (i.e. > 50% of the inoculated sites showing clear cell death) were counted and expressed as the mean percentage of total inoculations per plant.

### Plant treatments, coimmunoprecipitation and immunoblotting

For coimmunoprecipitation (co‐IP) and/or immunoblotting, N‐terminally tagged vectors GFP‐Stphot1, GFP‐Stphot2, RFP‐Stphot1, RFP‐Stphot1.KD, RFP‐Stphot2, cMYC‐StNRL1 and GFP‐StSWAP70 as well as control vectors RFP‐GUS and cMYC‐GUS were expressed with various combinations in *N. benthamiana* leaves using Agrobacterium‐mediated transformation. Proteins were extracted using GTEN buffer (10% (v/v) glycerol, 25 mM Tris–HCl (pH 7.5), 1 mM EDTA, 150 mM NaCl) with 10 mM dithiothreitol (DTT), protease inhibitor cocktail, 1 mM phenylmethyl sulphonyl fluoride (PMSF) and 0.2% Nonidet P‐40. The co‐IPs were performed using GFP‐trap magnetic beads (Chromotek, Planegg, Germany) at 4°C for 1 h on a thermomixer. Beads were washed three times using GTEN‐based wash buffer containing protease inhibitor mixture and 1 mM PMSF. Proteins were eluted by boiling beads in 2× SDS sample buffer (100 mM Tris–HCl, 4% SDS, 20% glycerol, 0.2% bromophenol blue and 200 mM DTT). The resulting samples were separated by sodium dodecyl sulphate–polyacrylamide gel electrophoresis (SDS‐PAGE) and transferred to nitrocellulose membranes. GFP, cMYC or mRFP fusion proteins were detected using appropriate antisera (Santa Cruz Biotechnology, Santa Cruz, CA, USA). Primary antibodies (monoclonal GFP antibody raised in mouse (sc‐9996; Santa Cruz Biotechnology)), 1 : 4000; monoclonal cMYC antibody raised in mouse (SC‐40; Santa Cruz Biotechnology), 1 : 500; polyclonal mRFP antibody raised in rat (5F8; Chromotek), 1 : 4,000 were incubated overnight at 4°C and secondary antibodies (anti‐mouse Ig‐HRP antibody (A9044; Sigma‐Aldrich, St Louis, MO, US)) or anti‐rat Ig‐HRP antibody (ab6836; Abcam, Cambridge, MA, USA) for 1 h at room temperature.

### *Phytophthora infestans* infection assay

*Phytophthora infestans* strain 88069 was grown on rye agar medium at 19°C for 2 wk before infections. The plates were flooded with 5 ml H_2_O and scraped with an L‐shaped spreader to release sporangia. The sporangial suspension was collected into a Falcon tube and spun at 1598 ***g*** for 5 min at 4°C. Sporangia were counted using a haemocytometer then adjusted to 50 000 sporangia ml^–1^ for virus‐induced gene silencing (VIGS) plant infection and was elevated to 80 000 sporangia ml^–1^ for agroinfiltrated *N. benthamiana* leaves. For infections, 10 µl droplets were inoculated onto the abaxial side of detached *N. benthamiana* leaves (four spots per leaf) kept on moist tissue in sealed boxes. The lesions were measured at 7–8 dpi and expressed as mean lesion diameter.

### Statistical analysis

For comparison of two different treatments, an unpaired, two‐sided Welch’s *t*‐test was conducted. Where three or more treatments were compared, a one‐way ANOVA with subsequent Tukey’s honest significant difference (HSD) *post hoc* test was performed. Boxplots were plotted in R using the package ggplot2 (v.3.3.5; https://ggplot2.tidyverse.org) (Wickham, 2016). Individual data points (blue spots), the mean (red spots) and the median (black bar within the boxes) are shown for each boxplot.

### Virus‐induced gene silencing

Orthologues for genes encoding phototropins, cryptochromes and F‐box/kelch repeat‐containing proteins were identified in *N. benthamiana* using the sequences available in the genome database on the SolGenomics website (ftp://ftp.solgenomics.net/genomes/Nicotiana_benthamiana/). VIGS constructs were made by cloning *c*. 300 bp PCR fragments of *Nbphot1* and *Nbphot2* into pBinary tobacco rattle virus (TRV) vectors (Liu *et al*., 2002) between *Hpa*I and *Eco*RI sites in the antisense orientation (Fig. S1). To generate double constructs to silence *Nbphot1* and *Nbphot2* simultaneously, cloned fragments for *Nbphot1* and *Nbphot2* were joined by using suitable primers in a PCR (Tables S1, S2). To silence genes encoding cryptochromes (CRY1, CRY1‐like, CRY2 and CRY3) and F‐box/keltch repeat proteins (ZTL1 and FKF1), a quadruple and double VIGS constructs was synthesized, respectively (GenScript, Table S2). A TRV construct expressing GFP was used as a control (Liu *et al*., 2002; McLellan *et al*., 2013). The two largest leaves of four‐leaf stage *N. benthamiana* plants were pressure infiltrated with *A. tumefaciens* strain LBA4404 containing a mixture of RNA1 (OD_600_ = 0.3) and each phot VIGS construct or the GFP control at OD_600_ = 0.5. Plants were used to perform assays or to check gene silencing levels by quantitative reverse transcriptase PCR (qRT‐PCR) after 3 wk.

### Gene expression analysis

*Nicotiana benthamiana* leaves were collected from VIGS plants and were snap frozen in liquid nitrogen. Total leaf RNA was extracted using an RNeasy plant kit (Qiagen) with on‐column DNA digestion following the manufacturer’s instructions. Two micrograms of RNA was used for first‐strand cDNA synthesis using an iScript™ Advanced cDNA synthesis kit (BioRad) according to the manufacturer’s instructions. Quantitative real‐time RT‐PCR was performed using SYBR Green (Applied Biosystems, Foster City, CA, USA) and run on StepOne real‐time PCR system (Applied Biosystems). Primer sequences are given in Table S1. Primers were designed outside the region targeted for silencing. Gene expression analysis was performed using the comparative Ct method, as described by Cikos *et al*. (2007).

### Kinase assay

To measure kinase activity of Stphot1 *in vitro*, leaf samples from *N. benthamiana* inoculated with RFP‐Stphot1 or RFP‐Stphot1.KD were harvested 2 d after agroinfiltration. RFP‐Stphot1 wildtype and the kinase‐dead mutant RFP‐Stphot1.KD were immunoprecipitated as described above. Samples were washed in GTEN high salt buffer (GTEN supplemented with 0.7% NP40 and 500 mM NaCl) twice after incubation with beads, followed by three washing steps with wash buffer (50 mM Tris pH 7.6, 10 mM MgCl_2_, 0.1% NP40 and 150 mM NaCl). Beads were resuspended in 25 µl reaction buffer (wash buffer + 100 mM ATP) and preincubated for 1 h at 30°C. The supernatants were discarded followed by a second incubation in 25 µl reaction buffer for 1 h at 30°C. Beads were separated and the formation of ADP in the supernatants was quantified with a Universal kinase assay kit (Abcam) following the manufacturer’s instructions. ADP was measured on a plate reader using a GFP filter (525 nm/580–640 nm). Fluorescence units were normalized to beads incubated with protein extract from untreated leaves.

## Results

### Phototropins act as susceptibility factors in the plant–*P. infestans* interaction

**Fig. 1:**
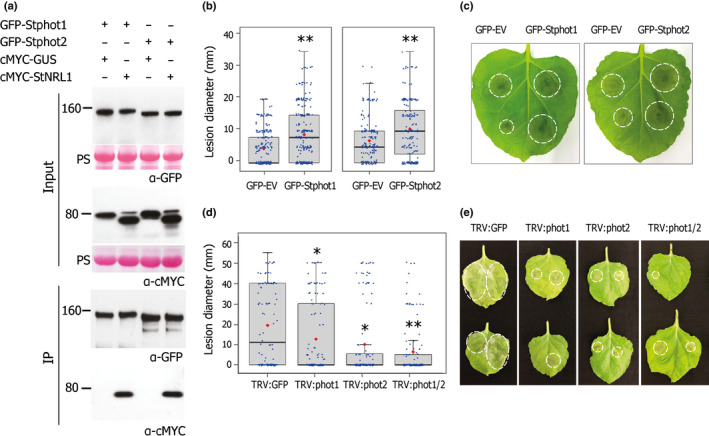
Stphot1 and Stphot2 both interact with StNRL1 and act as susceptibility factors. (a) The *in planta* interaction of Stphot1 and Stphot2 with StNRL1 was confirmed in *Nicotiana benthamiana* by coimmunoprecipitation. cMYC‐StNRL1 associated with both GFP‐Stphot1 and GFP‐Stphot2, whereas cMYC‐GUS did not. Constructs expressed in *N. benthamiana* leaves are indicated by a plus sign (+). Protein sizes are represented in kilodaltons (kDa) and protein loading is shown by Ponceau staining (PS). (b) Both GFP‐Stphot1 and GFP‐Stphot2, when transiently expressed in *N. benthamiana* leaves, enhanced *Phytophthora infestans* colonization compared to half leaves inoculated with *Agrobacterium* expressing GFP‐EV (Welch’s *t*‐test, **, *P* < 0.001; *n* = 240). Infection represented in box plots is measured as lesion diameter (mm) on inoculated leaves. (c) Representative leaf images for the graph in (b). (d) Leaves from VIGS plants silenced for *Nbphot1*, *Nbphot2* alone, or simultaneously *Nbphot1* and *Nbphot2* (TRV:phot1, TRV:phot2, TRV:phot1/2, respectively) when infected with *P. infestans* showed a reduction of lesion sizes compared to plants expressing TRV:GFP only (one‐way ANOVA with Tukey’s HSD *post‐hoc* test, *, *P* < 0.050; **, *P* < 0.001; *n* = 128). (e) Representative leaf images for the graph in (d). The results shown in (b, c) box plots are combinations of at least three independent experimental replicates, each comprising *c*. 3 leaves from *c*. 12 plants per repplicate. Error bars indicate ± SE. Blue dots represent individual datapoints in box plots; red dots indicate the mean values; and horizontal line is the median. Infection on leaves is indicated by dotted circles.

co‐IP was used to confirm the *in vivo* interactions between phots and StNRL1. GFP‐Stphot1 and GFP‐Stphot2 were transiently coexpressed with either cMYC‐GUS or cMYC‐StNRL1, using *Agrobacterium*‐mediated expression in *N*. *benthamiana*. Although all proteins were present in the relevant input samples, only cMYC‐StNRL1 was coimmunoprecipitated in the presence of GFP‐Stphot1 or GFP‐Stphot2, whereas the cMYC‐GUS control was not detected in the immunoprecipitated samples (Fig. 1a). Thus, StNRL1 interacts with both Stphot1 and Stphot2 *in planta*.

Since StNRL1 acts as a susceptibility (S) factor to promote *P. infestans* colonization (He *et al*., 2018), we investigated the contribution of Stphot1 and Stphot2 to disease susceptibility both by transiently overexpressing them and by silencing the orthologues in *N. benthamiana*. GFP‐EV was transiently expressed on one half of leaves and either GFP‐Stphot1 or GFP‐Stphot2 on the other half. Upon subsequent inoculation of each half leaf with *P. infestans* zoospores, lesions were measured to assess disease progression. Leaf halves infiltrated with either GFP‐Stphot1 or GFP‐Stphot2 showed significantly larger lesion sizes (Welch’s *t*‐test, *P* < 0.001; *n* = 240) compared to control halves expressing free GFP (Fig. 1b,c).

VIGS was used to knock down expression of *N. benthamiana* phot orthologues, *Nbphot1* and *Nbphot2*. Two independent silencing constructs were designed for each gene (TRV:phot1.V1 and V2; TRV:phot2.V1 and V2). To silence both genes together, cloned portions from *Nbphot1* and *Nbphot2* were combined into the TRV vector, designated TRV:phot1/2.V1 and V2 (Fig. S1a). qRT‐PCR was used to evaluate silencing levels for *Nbphot1* and *Nbphot2* in each of four independent biological replicates. The combined data show that the transcript levels of *Nbphot1* were reduced by 80% in plants expressing either TRV:phot1.V1 or TRV:phot1.V2 compared with plants expressing the TRV:GFP control, whereas there was no reduction in *Nbphot2* transcript level (Fig. S1b). By contrast, in plants expressing TRV:phot2.V1 or TRV:phot2.V2, the transcript levels for *Nbphot2* were reduced by 50%, whereas *Nbphot1* transcript abundance was unaltered. Expression of the dual silencing constructs TRV:phot1/2.V1 and TRV:phot1/2.V2 in *N. benthamiana* resulted in a decrease in transcript abundance for both genes, comparable to each of the individual silencing events (*Nbphot1* and *Nbphot2*; Fig. S1b). Since the two independent constructs each for silencing *Nbphot1* or *Nbphot2*, singly or in combination, resulted in similar levels of reduced transcript abundance, we chose one construct each for further analysis (Fig. S1c). Leaves from *N. benthamiana* plants expressing TRV:phot1, TRV:phot2 or the dual silencing construct TRV:phot1/2 were inoculated with *P. infestans* sporangia. Silencing of either *Nbphot1* or *Nbphot2* resulted in a significant reduction in *P. infestans* leaf colonization, with significantly smaller lesion sizes (one‐way ANOVA with Tukey’s HSD *post‐hoc* test, *P* < 0.050, *n* = 128) compared with the TRV:GFP control plants (Fig. 1d,e).

These results reveal that phot1 and phot2 expression enhance susceptibility to *P. infestans* infection, whereas silencing of each restricts *P. infestans* infection. This indicates that, similar to StNRL1 (Yang *et al*., 2016; He *et al*., 2018), phots from potato and *N. benthamiana* act as S factors, in that they are required for efficient infection. Moreover, simultaneous silencing of both *Nbphot1* and *Nbphot2* showed a further reduction in host colonization (Fig. 1d,e), suggesting that phot1 and phot2 each play nonredundant (independent) roles as S factors.

### Phototropin 1 shows NRL1‐ and BL‐dependent suppression of INF1‐triggered cell death

We have shown previously that the Pi02860 effector target, StNRL1, suppresses the ICD immune response (Yang *et al*., 2016). To investigate whether ICD suppression is a general property of the NRL family, we cloned potato NPH3 and RPT2, which are among the best characterized NRL family members involved in phot signalling, and tested whether their expression also suppressed ICD to enhance *P. infestans* infection. INF1 was coexpressed with GFP‐StNPH3, GFP‐StRPT2, GFP‐StNRL1 or GFP‐EV. Where GFP‐StNRL1 suppressed ICD compared to the GFP‐EV control, GFP‐StNPH3 and GFP‐StRPT2 had no effect on the cell death response (Fig. S2a). For *P. infestans* infection assays, GFP‐StNPH3 and GFP‐StRPT2 were transiently expressed in *N. benthamiana* leaves 1 d before infections. When leaves were challenged with *P. infestans*, GFP‐StNPH3‐ and GFP‐StRPT2‐expressing sites showed similar infection levels as the GFP‐EV control, indicating that, unlike StNRL1 (Yang *et al*., 2016), StNPH3 and StRPT2 did not enhance *P. infestans* colonization (Fig. S2b). Both GFP‐StNPH3 and GFP‐StRPT2 were expressed as stable fusion proteins (Fig. S2c).

**Fig. 2:**
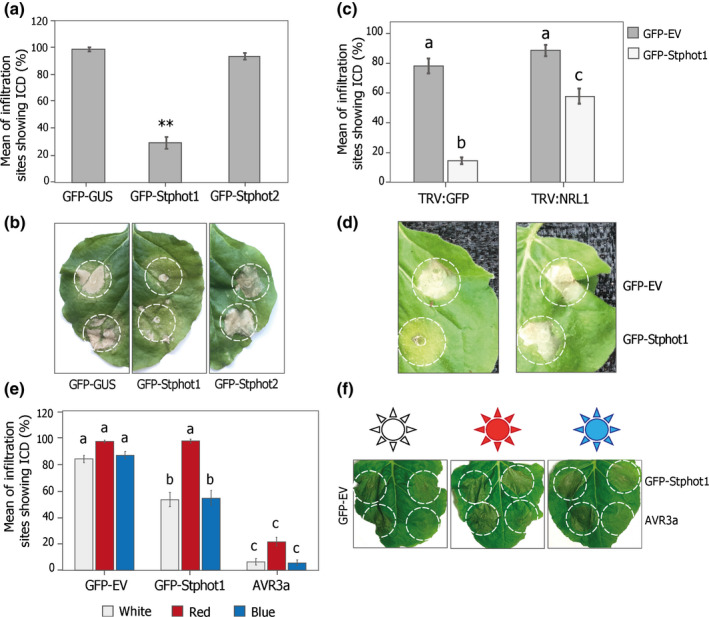
Stphot1, but not Stphot2, suppresses INF1‐triggered cell death (ICD). (a, b) Transient expression of GFP‐Stphot1 in *Nicotiana benthamiana* leaves shows suppression of ICD compared to GFP‐EV control (one‐way ANOVA with Tukey’s HSD *post‐hoc* test, **, *P* < 0.001; *n* = 52). Unlike GFP‐Stphot1, GFP‐Stphot2 does not alter ICD levels. (b) Example leaves for the graph in (a) showing ICD suppression by GFP‐Stphot1. (c) The ability of GFP‐Stphot1 to suppress ICD is greatly reduced in *NRL1*‐silenced plants (expressing TRV:NRL1) compared to TRV:GFP control plants (one‐way ANOVA with Holms–Sidak adjustment, *P* < 0.001; *n* = 160, and significant differences are indicated by letters). Graphs were plotted using combined data of four independent experimental replicates, consisting of at least 40 inoculations each. (d) Example leaves for the graph in (c) showing ICD suppression by GFP‐Stphot1. (e) GFP‐Stphot1 when coexpressed with INF1 suppresses ICD under white and blue light conditions but not in red, confirming that blue‐light‐mediated activation of Stphot1 is required for its role in suppression of ICD. Different wavelengths of lights were provided under 16 h : 8 h light : dark cycles. Graph represents a combination of four experimental replicates, consisting of at least 10 plants and four leaves per plant each. A significant difference is denoted with letters (one‐way ANOVA with Tukey’s HSD *post‐hoc* test, *P* < 0.001, *n* = 144). (f) Example leaf images for the graph in (e) show the ICD response on the left sides of the leaves infiltrated with a combination of GFP‐EV control and INF1. AVR3a when coexpressed with INF1 (bottom right) can suppress ICD under all three light conditions, whereas GFP‐Stphot1 (top right) supresses ICD under white and blue LEDs but not in red.

As phots act upstream of NRL proteins (Christie *et al*., 2018), and both Stphot1 and Stphot2 interact with StNRL1 (Fig. 1a), we investigated whether they are able to suppress ICD. GFP‐Stphot1, GFP‐Stphot2 or GFP‐EV as a control were coexpressed with INF1 and, at 5 dpi, ICD occurrence on leaves was measured as a percentage of inoculation sites showing a cell death response. GFP‐Stphot1 coexpressed with INF1 significantly reduced ICD compared to the GFP control (ANOVA, *P* < 0.001; *n* = 52). Surprisingly, GFP‐Stphot2 had no effect on ICD (Fig. 2a,b). This demonstrates that the ability of StNRL1 to suppress ICD is shared only with Stphot1, but not Stphot2.

To determine whether Stphot1 suppression of ICD is NRL1‐dependent we coexpressed INF1 with either GFP‐EV or GFP‐Stphot1 in *N. benthamiana* plants where *NbNRL1* is silenced, using a previously described TRV:NRL1.V1 VIGS construct (He *et al*., 2018). The ability of Stphot1 to suppress ICD was significantly reduced in *N. benthamiana* plants expressing TRV:NRL1.V1 (one‐way ANOVA with Tukey’s HSD *post‐hoc* test, *P* < 0.001; *n* = 40) compared to TRV:GFP control plants (Fig. 2c,d). Given that the silencing does not completely attenuate *NbNRL1* levels, this result provides strong evidence that the ability of Stphot1 to suppress ICD is NRL1‐dependent.

Phot1‐mediated ICD suppression was observed under white (WL) and blue light conditions (BL) but not in red (RL), while the control effector AVR3a suppressed the ICD response irrespective of light wavelength (Fig. 2e,f). Hence suppression of ICD by Stphot1 is dependent on its activation downstream of BL.

### Stphot1 reduces StSWAP70 abundance

We previously showed that transient expression of StNRL1, or of the *P. infestans* RXLR effector Pi02860, causes 26S proteasome‐mediated degradation of StSWAP70, leading to ICD suppression (He *et al*., 2018). We therefore investigated the effect of Stphot1 overexpression on StSWAP70 protein abundance. GFP‐StSWAP70 was coexpressed with a range of different proteins: RFP‐GUS and cMYC‐GUS, as a control; with RFP‐GUS and cMYC‐StNRL1; with RFP‐GUS and cMYC‐Pi02860; with cMYC‐StNRL1 and cMYC‐Pi02860; with RFP‐Stphot1 and cMYC‐GUS; or with RFP‐Stphot1 and cMYC‐StNRL1 (Fig. S3). As expected, cMYC‐StNRL1 coexpression reduced GFP‐StSWAP70 protein levels. Moreover, effector cMYC‐Pi02860, expressed alone or in combination with cMYC‐StNRL1, caused further reduction in StSWAP70 protein abundance, again as anticipated (He *et al*., 2018). Coexpression of RFP‐Stphot1 and cMYC‐GUS with GFP‐StSWAP70 resulted in a moderate reduction of StSWAP70 protein levels but the combination of RFP‐Stphot1 and cMYC‐StNRL1 resulted in further reduction in StSWAP70, similar to the levels observed for StSWAP70 when coexpressed with cMYC‐StNRL1 and cMYC‐Pi02860 (Fig. S3). A reduction in StSWAP70 protein abundance mediated by its coexpression with Stphot1 is consistent with the ability of Stphot1 to suppress ICD. As Stphot2 failed to suppress ICD, we predicted that it would also fail to stimulate GFP‐StSWAP70 degradation.

**Fig. 3:**
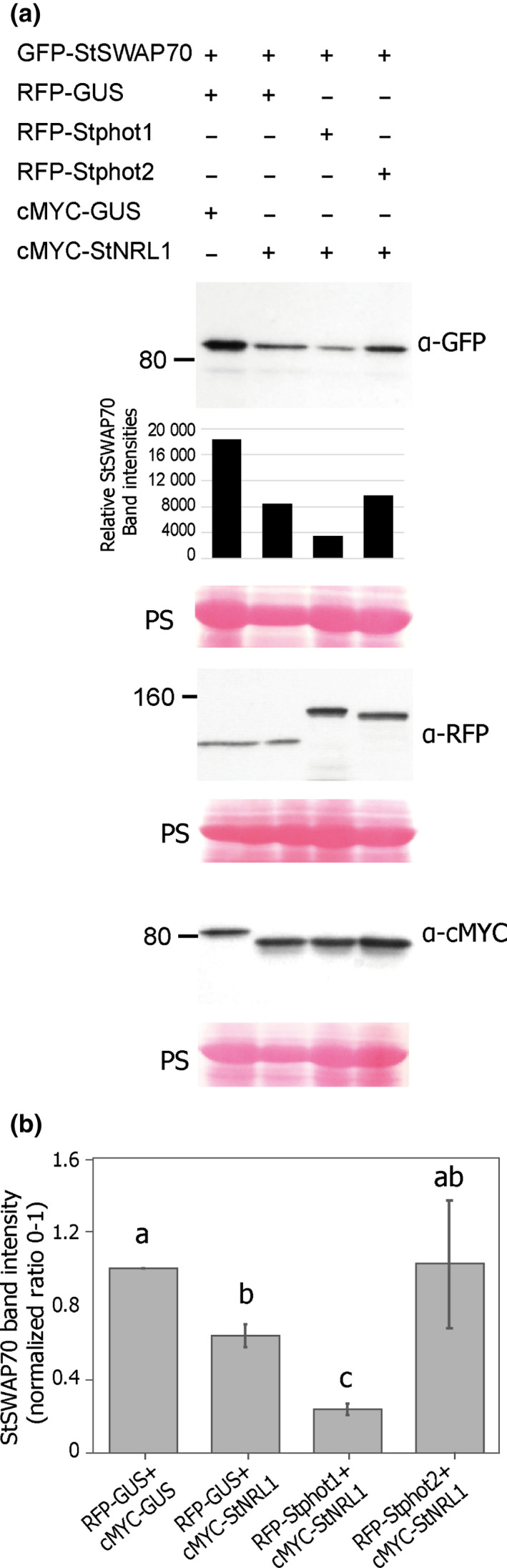
Stphot1, but not Stphot2, reduces StSWAP70 abundance. (a) Further immunoblot analysis shows that, in the presence of cMYC‐StNRL1, reduced GFP‐StSWAP70 abundance is stimulated specifically by coexpression with RFP‐Stphot1 and not RFP‐Stphot2 or the RFP‐GUS control. Constructs expressed in *Nicotiana benthamiana* leaves are indicated by a plus sign (+). Protein sizes are represented in kilodaltons (kDa) and protein loading is shown by Ponceau staining (PS). The graphs below GFP‐StSWAP70 show relative intensity of bands. (b) Graph showing the mean StSWAP70 band intensities normalized to the control (expressed with RFP‐GUS and cMYC‐GUS, which was given a value of 1) from three independent replicate experiments of the immunoblots shown in (a) and Supporting Information Fig. S4. GFP‐StSWAP70 protein levels were reduced when coexpressed with RFP‐Stphot1 and cMYC‐StNRL1 (one‐way ANOVA, *P* < 0.050) but not with RFP‐Stphot2 or cMYC‐StNRL1, which were instead similar to the RFP‐GUS and cMYC‐StNRL1 controls. Error bars indicate SE and letters on the graphs denote statistically significant differences (one‐way ANOVA).

To test this hypothesis, we performed a series of immunoblots in which GFP‐StSWAP70 was coexpressed with RFP‐GUS and cMYC‐GUS as a control; with RFP‐GUS and cMYC‐StNRL1; with RFP‐Stphot1 and cMYC‐StNRL1; or with RFP‐Stphot2 and cMYC‐StNRL1 (Fig. 3a). As anticipated, GFP‐StSWAP70 abundance was reduced following coexpression with cMYC‐StNRL1 and RFP‐GUS, compared to the control cMYC‐GUS and RFP‐GUS. GFP‐StSWAP70 abundance was further reduced when coexpressed with RFP‐Stphot1 and cMYC‐StNRL1 (Figs 3a,b, S4). However, the combination of RFP‐Stphot2 and cMYC‐StNRL1 showed levels of GFP‐StSWAP70 that were either comparable to coexpression with RFP‐GUS and cMYC‐StNRL1 (Figs 3a,b, S4), or were even similar to levels observed following coexpression with the RFP‐GUS and cMYC‐GUS control (Figs 3b, S4b). This indicates that Stphot1 in conjunction with StNRL1 causes StSWAP70 degradation, consistent with ICD suppression, whereas Stphot2 does not. Interestingly, coexpression of Stphot2 diminished the impact of StNRL1 on StSWAP70 levels in one replicate (Fig. S4b), perhaps suggesting it can antagonize StSWAP70 turnover.

### Stphot1 kinase activity is required for suppression of ICD and to reduce StSWAP70 abundance

Phots undergo BL‐dependent autophosphorylation, which is an absolute requirement for phot‐mediated physiological responses (Inoue *et al*., 2008). In the Arabidopsis phot1 kinase domain, substitution of an aspartate residue (806) with an asparagine results in a kinase‐dead form by preventing binding of Mg^2+^ for phosphate transfer (Hanks & Hunter, 1995). As a consequence of this mutation, abolition of phot1 autophosphorylation and its mediated responses has been reported (Christie *et al*., 2002; Inoue *et al*., 2008; Christie & Murphy, 2013). To investigate the role of kinase activity in suppression of ICD and promotion of *P. infestans* infection, we located the respective aspartate residue in Stphot1 and mutated it to asparagine, generating a mutant form Stphot1.D832N, which we refer to as RFP‐Stphot1.KD (kinase‐dead). The abolition of kinase activity in Stphot1.KD was confirmed by an *in vitro* kinase assay. RFP‐Stphot1 and RFP‐Stphot1.KD expressed in *N. benthamiana* were immunoprecipitated from leaf protein extracted using RFP‐coupled beads which were incubated with ATP. Measurement of ADP present in the samples indicated RFP‐Stphot1 autophosphorylation, resulting in ADP formation from ATP, while no ADP was detected with the kinase‐dead mutant RFP‐Stphot1.KD (Fig. S5a). The inability of RFP‐Stphot1.KD to undergo autophosphorylation was further confirmed by a gel mobility shift assay. Protein samples were separated on a low percentage acrylamide (6.5%) gel to achieve better separation and indication of size differences. RFP‐Stphot1 extracted from leaves exposed to 16 h light showed an increased electrophoretic mobility shift due to receptor autophosphorylation compared to 16 h dark‐treated samples. By contrast, RFP‐Stphot1.KD failed to show this mobility shift in the light compared to 16 h dark treatment, confirming a lack of autophosphorylation (Fig. S5b).

**Fig. 4:**
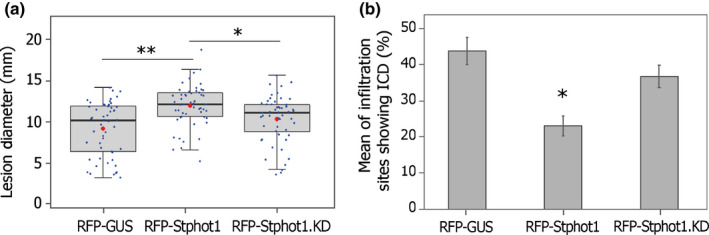
Stphot1 kinase activity is required to enhance *Phytophthora infestans* colonization and suppress ICD. (a) Transient expression of RFP‐Stphot1 promotes *P. infestans* infection, whereas when amino acid residue D832 in the Stphot1 kinase domain was mutated to asparagine (Stphot1.KD), it can no longer enhance *P. infestans* colonization on *Nicotiana benthamiana* leaves (one‐way ANOVA with Tukey’s HSD *post‐hoc* test, **, *P* < 0.001; *, *P* < 0.05; *n* = 104). Blue dots are individual datapoints; red dots indicate mean; and horizontal lines are the median. (b) Graphs showing that the Stphot1 kinase‐dead mutant RFP‐Stphot1.KD fails to suppress ICD when coinfiltrated with INF1 in *N. benthamiana,* compared to wild‐type RFP‐Stphot1 (one‐way ANOVA, *, *P* < 0.05; *n* = 160). The graphs in (a) and (b) represent combined data of three independent experimental replicates.

We next tested the effect of the kinase‐dead mutant upon disease progression. RFP‐Stphot1 or RFP‐Stphot1.KD were expressed on one half of an *N. benthamiana* leaf with RFP‐EV control on the other, and each half was inoculated with *P. infestans* zoospores. The area of the leaves expressing RFP‐Stphot1.KD showed lesion sizes comparable to the RFP‐EV while significantly larger lesions were observed following expression of RFP‐Stphot1 WT as expected (Fig. 4a), indicating that the mutant failed to enhance infection. To test the effect of this mutation on ICD suppression, we transiently coexpressed RFP‐GUS, RFP‐Stphot1 or RFP‐Stphot1.KD with INF1 in *N. benthamiana* leaves. The ability of RFP‐Stphot1.KD to suppress ICD was significantly attenuated (one‐way ANOVA with Tukey’s HSD *post‐hoc* test, *P* < 0.001) compared with the RFP‐Stphot1 WT (Fig. 4b).

**Fig. 5:**
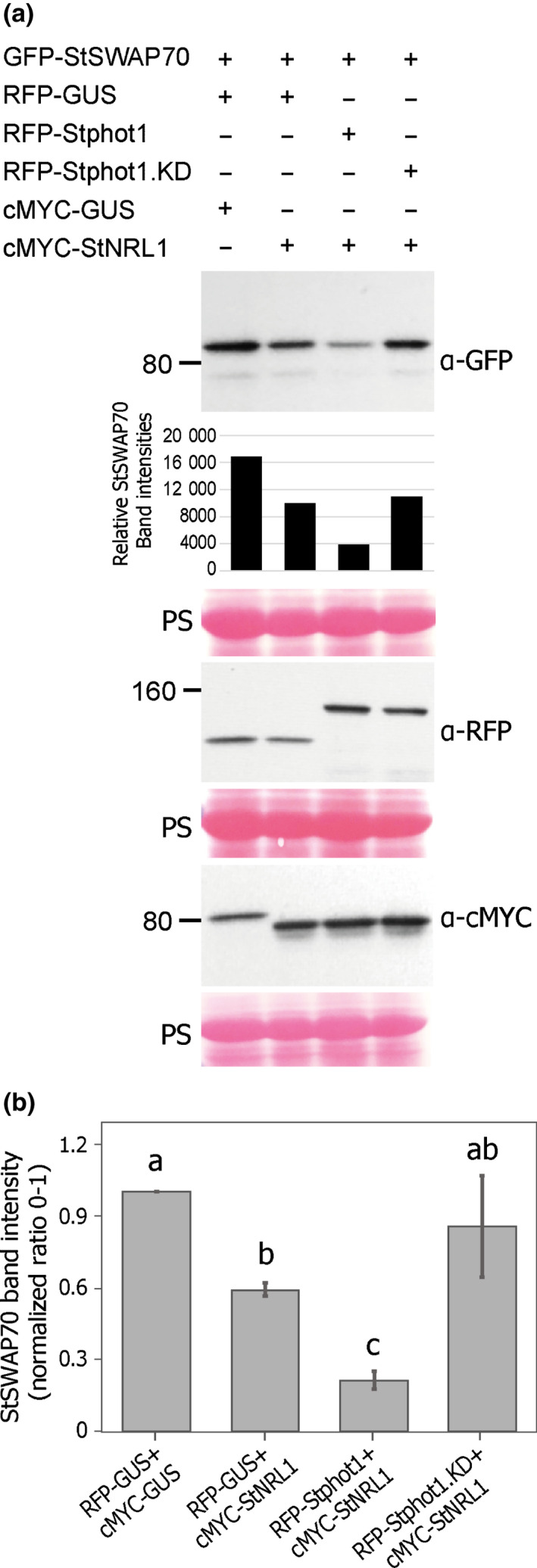
Stphot1 kinase activity is required to reduce StSWAP70 abundance. (a) Immunoblots showing that the GFP‐StSWAP70 protein level is reduced when coexpressed with either RFP‐Stphot1+cMYC‐GUS or RFP‐Stphot1+cMYC‐StNRL1 but not with the kinase‐dead mutant RFP‐Stphot1.KD and cMYC‐StNRL1. Constructs expressed in *Nicotiana benthamiana* leaves are indicated by a plus sign (+). Protein sizes are represented in kilodaltons (kDa) and protein loading is shown by Ponceau staining (PS). The graph below shows the intensity of bands analysed. (b) Graph showing the mean StSWAP70 band intensity ratios (0–1) normalized to GUS controls (which were given a value of 1) from three independent replicate experiments of immunoblots in (a) and Supporting Information Fig. S6. The StSWAP70 protein levels in the presence of cMYC‐StNRL1 were significantly reduced when coexpressed with wild‐type RFP‐Stphot1 compared to RFP‐GUS control (one‐way ANOVA, *P* < 0.05) but not with the kinase‐dead mutant RFP‐Stphot1.KD. Error bars indicate SE and letters on the graphs denote statistically significant differences (one‐way ANOVA).

We compared the ability of the Stphot1.KD mutation to reduce StSWAP70 abundance with the WT Stphot1. As already seen (Figs 3, S4), GFP‐StSWAP70 protein levels were reduced by coexpression with cMYC‐StNRL1, and the combined expression of cMYC‐StNRL1 and RFP‐Stphot1 caused a further reduction in StSWAP70 (Figs 5a,b, S6). However, coexpression of RFP‐Stphot1.KD with cMYC‐StNRL1 failed to further reduce GFP‐StSWAP70 abundance (Figs 5a,b, S6).

### Blue light enhances *P*. *infestans* colonization

**Fig. 6:**
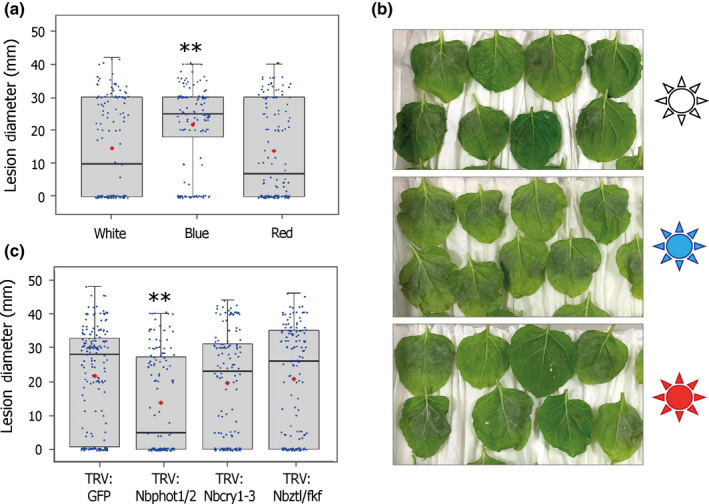
Blue light causes susceptibility to *Phytophthora infestans* through Stphot1/2. (a) *Nicotiana benthamiana* leaves inoculated with *P. infestans* spores developed bigger lesions in blue light conditions compared to red or white LEDs (one‐way ANOVA with Tukey’s HSD *post‐hoc* test, **, *P* < 0.001; *n* = 132). (b) Representative leaf images for the graph in (a) showing *P. infestans* growth under different light conditions tested. (c) Boxplots showing lesion sizes on VIGS plants silenced for known blue light receptors *Nbphot1/2*, *NbCry1‐3* and *Nbztl/fkf* compared to TRV:GFP control. While quadruple silencing *Nbcry1‐3* and simultaneous silencing of *Nbztl1* and *Nbfkf1* have no effect on *P. infestans* infection, silencing of *Nbphot1* and *Nbphot2* together results in significant reduction in infections (one‐way ANOVA with Tukey’s HSD *post‐hoc* test, **, *P* < 0.001; *n* = 150). Error bars indicate ± SE. Blue dots represent individual datapoints in box plots; red dots indicate the mean values; and horizontal line is the median. The graphs in (a, c) represent combined data of four independent experimental replicates.

To establish the influence of monochromatic BL on *P*. *infestans* growth, we grew *P*. *infestans* cultures on rye agar for 16 h under BL, RL or WL wavelength LEDs, followed by 8 h of darkness. Measurement of growth diameters on day 10 showed a similar growth pattern under WL and BL, whereas RL enhanced hyphal growth on the plates (Fig. S7a,b). By contrast, when inoculated onto *N. benthamiana* leaves, followed by 7–8 d of similar light conditions, larger lesions and more homogenous infection were clearly evident under BL compared to RL or WL, indicating that BL enhances late blight disease susceptibility (Fig. 6a,b).

The enhanced susceptibility under BL conditions could be explained by the earlier observations that phot1 and phot2 act as S factors. However, BL is also perceived by additional receptors: cryptochromes (Cashmore *et al*., 1999) and the KFB subfamily (Suetsugu & Wada, 2013). To establish whether the enhanced susceptibility on BL is likely to be due to phot receptors alone, we developed TRV VIGS constructs that simultaneously silence all three *Nbcry* genes in *N. benthamiana* (and the closely related *Nbcry1‐like* gene) by 80% (Fig. S8a,b). In addition, TRV VIGS constructs were designed that silenced both genes encoding F‐box proteins (*Nbztl1* and *Nbfkf1*) in *N. benthamiana*, which reduced transcript levels by 70% (Fig. S8c,d). Whereas, as already observed (Fig. 1e,f), *Nbphot1/2* double silenced plants significantly reduced *P. infestans* colonization, silencing of *Nbcry* genes or of *Nbztl1* and *Nbfkf1* had no impact on the level of infection (Fig. 6c).

## Discussion

In this study, we show that expression of the BL receptors Stphot1 and Stphot2 promote susceptibility to *P. infestans*, probably through different mechanisms. Independent silencing of *Nbphot1* or *Nbphot2* showed that each is required for full susceptibility to late blight and they can thus be regarded as S factors. Combined silencing of both genes resulted in further reduced susceptibility (Fig. 1), indicating that they play nonredundant roles as S factors. Indeed, whereas Stphot1 suppresses ICD, Stphot2 does not (Fig. 2). Moreover, coexpression of Stphot1 with StNRL1 led to reduced abundance of the positive immune regulator StSWAP70, whereas overexpression of Stphot2 did not (Fig. 3), underlining their distinct contributions to susceptibility. The reduction in StSWAP70 protein levels when coexpressed with StNRL1 was shown previously to be prevented by the inhibitor MG132, indicating that StNRL1 acts as a ubiquitin E3 ligase to facilitate proteasome‐mediated degradation of StSWAP70 (He *et al*., 2018). It is likely that Stphot2 interferes with other immune processes, or it may promote other physiological responses that are beneficial to a pathogen. It has been noted that phot2 cooperates with cry2 to regulate the stability of some resistance proteins (Jeong *et al*., 2010), demonstrating that phot2 and phot1 can act independently and that phot2 can regulate immunity through a different mechanism.

In control of responses such as phototropism, NRL family members such as NPH3 and RPT2 are proposed to work downstream of phot1 and their phosphorylation status changes upon phot1 activation (Haga *et al*., 2015; Sullivan *et al*., 2019). However, overexpressing StNPH3 and StRPT2 had no apparent effect on late blight disease progression or host immunity (Fig. S2) and these NRL family members may thus be more exclusively involved in modulating phot‐mediated physiological responses that regulate growth and development. By contrast, suppression of ICD by Stphot1 was dependent on its signal‐transducing NRL family member, StNRL1 (Fig. 2), which is also an S factor and a target of *P. infestans* RXLR effector Pi02860 (He *et al*., 2018).

**Fig. 7:**
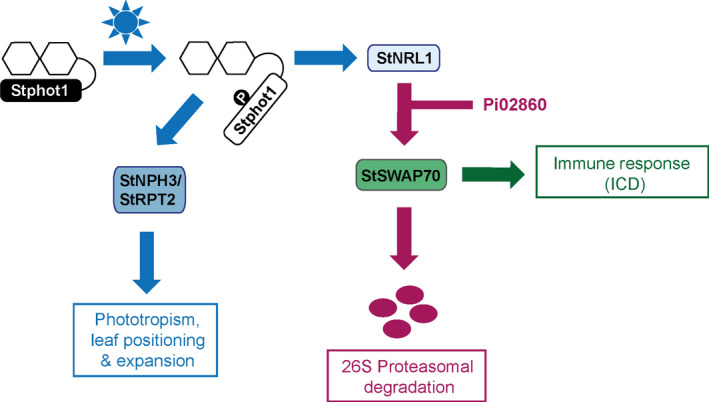
Stphot1 negatively regulates plant immunity. Phot1 undergoes autophosphorylation upon sensing blue light (blue arrows), leading to the direct activation of NRL family members, such as NPH3 and RPT2, which regulate several physiological responses such as phototropism, leaf flattening and positioning (Christie *et al*., 2018). The kinase activity of Stphot1 also activates StNRL1, which suppresses (purple arrows) ICD by targeting StSWAP70, a positive regulator of immunity (green arrow), for its proteasome‐mediated degradation. *Phytophthora infestans* effector Pi02860, as previously shown (He *et al*., 2018), enhances the ability of StNRL1 to further reduce StSWAP70 protein abundance.

BL‐triggered autophosphorylation of phots is an absolute requirement for phot‐mediated physiological responses (Christie *et al*., 2015). In keeping with this, an Stphot1 kinase‐dead mutant (Stphot1.KD) was unable to enhance *P. infestans* colonization or suppress ICD (Fig. 4), and also failed to reduce StSWAP70 abundance when coexpressed with StNRL1 (Fig. 5). Taken together, our results indicate that BL activation of Stphot1 kinase activity triggers an endogenous pathway that suppresses ICD, through the activity of the ubiquitin E3 ligase StNRL1, leading to the degradation of StSWAP70 (Fig. 7). Further work is needed to precisely determine how Stphot1 mediates degradation of StSWAP70 via StNRL1. However, recently, Arabidopsis phots have been reported to activate NPH3 by direct phosphorylation of the 14‐3‐3‐binding RxSxS motif at its C‐terminus (Sullivan *et al*., 2021). Given that StNRL1 also possesses a C‐terminal RxSxS motif, it is thus possible that direct phosphorylation of StNLR1 by Stphot1 leads to activation of the former. It is interesting that the pathogen *P. infestans* targets this endogenous immune‐suppressive pathway with the RXLR effector Pi02860 at the level of StNRL1 (He *et al*., 2018), rather than at the level of Stphot1. It is perhaps possible, and beneficial, for the pathogen to be able to specifically enhance StNRL1 activity to degrade StSWAP70 without perturbing the wider upstream functions of Stphot1 in association with other NRLs.

Finally, we show that BL enhances colonization of *N. benthamiana* by *P. infestans* (Fig. 6). As BL does not alter growth of the pathogen *in vitro* (Fig. S8), we infer that the observed enhancement of infection is the result of BL altering plant susceptibility. By contrast, we observed that, whereas RL enhanced growth of *P. infestans in vitro* compared to WL or BL (Fig. S7), colonization of leaves was not enhanced under RL (Fig. 6). This could be due to the fact that RL also promotes plant immunity. Indeed, although not statistically significant, RL appeared to enhance the ICD immune response (Fig. 2). RL activates the photoreceptor phyB, which is associated with positive regulation of the defence hormones jasmonic acid (JA) and salicylic acid (SA) (Ballaré & Pierek, 2017). Inactivation of phyB by high relative levels of far red (FR) light leads to the shade‐avoidance syndrome (SAS): plant growth to maximize light capture. SAS is associated with suppression of plant immunity, in part through the inactivation of phyB (Fernandez‐Milmanda *et al*., 2020; Fernandez‐Milmanda & Ballaré, 2021). Perception of BL by phots, promoting growth towards gaps in the canopy, and activation of CRYs by the low BL occurring under shade conditions, are both regarded as contributing to SAS, and thus may potentially act negatively on immunity (Ballaré & Pierek, 2017; Fernandez‐Milmanda & Ballaré, 2021). Interestingly, whereas silencing *Nbphots* reduced late blight susceptibility, silencing of *CRY* and F‐box BL receptors had no discernible effect on disease development. Enhanced late blight infection under BL conditions is thus more probable due to the action of phots. In addition to playing a role in SAS by contributing to phototropism, phot1 also promotes chloroplast accumulation movement via NCH1 (Suetsugu *et al*., 2016). Chloroplasts have been shown to navigate to nuclei in an actin‐dependent manner in response to PAMP perception (Caplan *et al*., 2015; Kumar *et al*., 2018; Ding *et al*., 2019). Chloroplast accumulation at nuclei during immune responses results in H_2_O_2_ transfer from the plastids to the nucleus to trigger cell death (Caplan *et al*., 2015). Interestingly, SWAP70, which is conserved in animals and plants, forms oligomers that are involved in tethering and stabilizing F‐actin to target membranes, raising the possibility that it is involved in chloroplast movement during immunity (Baranov *et al*., 2019). Further work is needed to investigate whether BL activation of StNRL1 by Stphot1, leading to degradation of StSWAP70, has any negative, or antagonistic, impact upon immune‐associated chloroplast movement.

In conclusion, we reveal that BL‐mediated activation of Stphot1 triggers an endogenous pathway that negatively regulates immunity. Further work is needed to precisely understand the crosstalk between light perception through various light receptors and innate immune responses. However, it is evident that the *P. infestans* effector Pi02860 exploits a key regulatory node by targeting StNRL1, downstream of Stphot1, to promote susceptibility. Unravelling the roles of light perception in regulating plant immunity is essential for developing novel strategies towards disease resistance and improved crop production without adversely affecting light‐regulated growth and development.

## Author contributions

PRJB, SN, EMG, QH and JMC designed the research. SN, QH, FT, HQ and QS performed the research. SN, PRJB, QH, FT and JP analysed and interpreted the data. SN and PRJB wrote the manuscript with input from all authors.

## Supporting information


**Fig. S1** Virus‐induced gene silencing (VIGS) of *Nbphot* genes in *Nicotiana benthamiana*.
**Fig. S2** StNPH3 and StRPT2 have no effect on ICD and *Phytophthora infestans* colonization.
**Fig. S3** Similar to StNRL1 and Pi02860, Stphot1 coexpression also reduces the abundance of StSWAP70.
**Fig. S4** Replicate immunoblots of Fig. 3(a).
**Fig. S5** Stphot1 mutant Stphot1.D832N is kinase‐dead (Stphot1.KD).
**Fig. S6** Replicate immunoblots of Fig. 5(a).
**Fig. S7** *In vitro* growth of *Phytophthora infestans* under various light conditions.
**Fig. S8** Silencing of *CRYs*, *ZTL* and *FKF* genes in *Nicotiana benthamiana*.
Please note: Wiley Blackwell are not responsible for the content or functionality of any Supporting Information supplied by the authors. Any queries (other than missing material) should be directed to the *New Phytologist* Central Office.

## Data Availability

The data that support the findings of this study are available from the corresponding author upon reasonable request.
