# Supplementary material for: Blue‐light receptor phototropin 1 suppresses immunity to promote Phytophthora infestans infection
Source: New Phytol. 2022 Jan 8;233(5):2282–93. doi: 10.1111/nph.17929 (PMC9255860; doi:10.1111/nph.17929)
Supplement: Supplementary file 1 — Fig. S1 Virus‐induced gene silencing (VIGS) of Nbphot genes in Nicotiana benthamiana. Fig. S2 StNPH3 and StRPT2 have no effect on ICD and Phytophthora infestans colonization. Fig. S3 Similar to StNRL1 and Pi02860, Stphot1 coexpression also reduces the abundance of StSWAP70. Fig. S4 Replicate immunoblots of Fig. 3(a). Fig. S5 Stphot1 mutant Stphot1.D832N is kinase‐dead (Stphot1.KD). Fig. S6 Replicate immunoblots of Fig. 5(a). Fig. S7 In vitro growth of Phytophthora infestans under various light conditions. Fig. S8 Silencing of CRYs, ZTL and FKF genes in Nicotiana benthamiana. Please note: Wiley Blackwell are not responsible for the content or functionality of any Supporting Information supplied by the authors. Any queries (other than missing material) should be directed to the New Phytologist Central Office. [file NPH-233-2282-s001.pdf]

***New Phytologist* Supporting Information**

Article title: **Blue-light receptor phototropin 1 suppresses immunity to promote *Phytophthora infestans* infection**

Authors: Shaista Naqvi, Qin He, Franziska Trusch, Huishan Qiu, Jasmine Pham, Qingguo Sun, John M Christie, Eleanor M Gilroy, Paul RJ Birch

Article acceptance date: 04 November 2021

The following Supporting Information is available for this article:

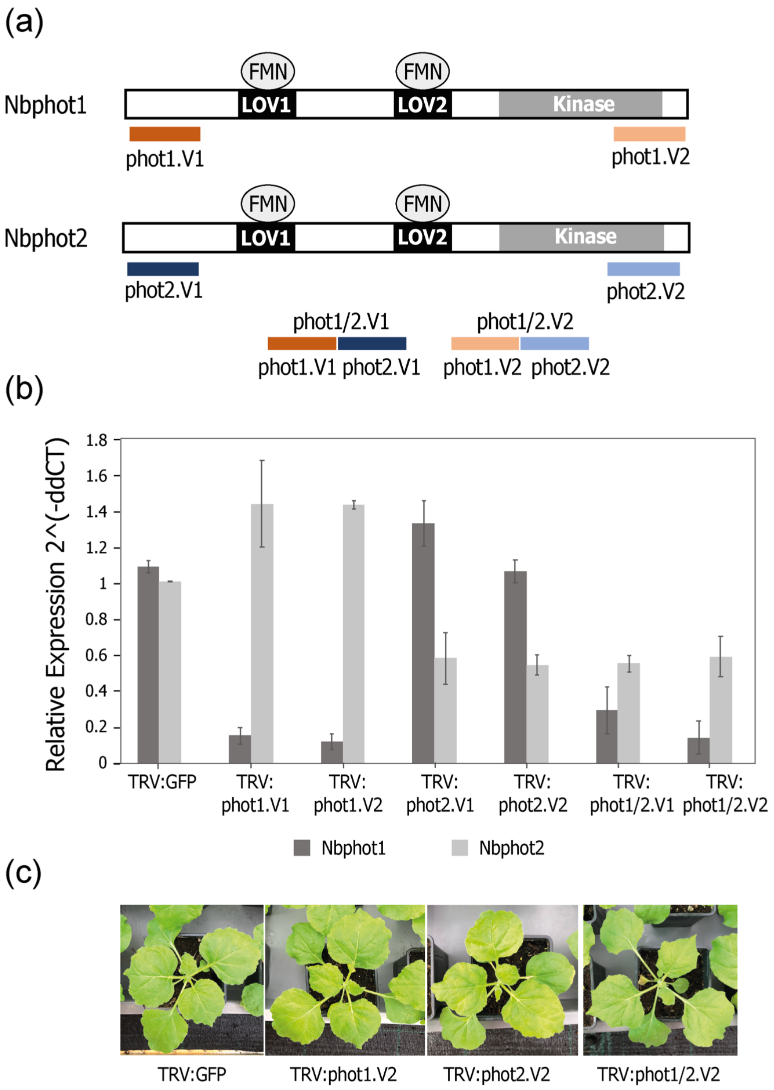

Fig. S1 Virus induced gene silencing (VIGS) of *Nbphot* genes in *N. benthamiana.*

(a) Schematic diagram for the *Nbphot1* and *Nbphot2* genes showing the positions of two LOV domains LOV1 and LOV2 and a kinase domain. The areas used to generate the VIGS silencing constructs are shown in colours below. Two fragments joined for each *Nbphot1* and *Nbphot2* sequences to achieve double silencing constructs are shown below the diagrams. **(b)** Graphs showing qRT-PCR analysis on leaves from silenced *N. benthamiana* plants indicates an 80 % reduction in *Nbphot1* transcript levels in TRV:Nbphot1.V1 and V2 and a 50 % reduction of *Nbphot2* transcript in plants expressing TRV:Nbphot2.V1 and V2 constructs. The double *Nbphot1/2* contructs TRV:Nbphot1/2.V1 and V2 showed reduction of both transcripts simultaneously. TRV:GFP used as a control to normalise the gene expression. The analysis was performed using the 2ddCt method and the error bars indicate +/- SE. The data represented is a combination of four independent experiments. **(c)** Representative plants expressing TRV constructs as indicated for silencing *Nbphot1, Nbphot2* and both *Nbphot1/2*. VIGS of *Nbphots* in *N. benthamiana* to the levels presented in graph (b) did not show any adverse effects on growth phenotypes compared to the TRV:GFP control.

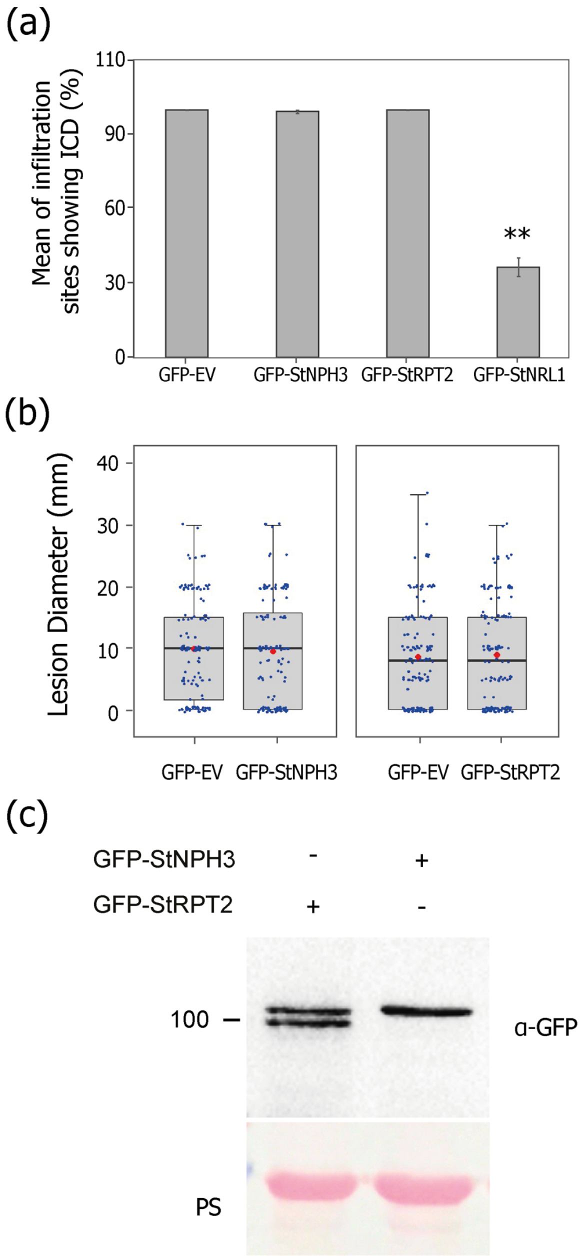

Fig. S2 StNPH3 and StRPT2 have no effect on ICD and *P. infestans* colonisation.

(a) Transient expression of GFP-StNPH3 or GFP-StRPT2 in *N. benthamiana* shows no suppression ICD while GFP-StNRL1 suppresses it as expected (one-way ANOVA with Tukey's HSD Post-Hoc test, **$p<0.001$; $n=120$). **(b)** GFP-StNPH3 and GFP-StRPT2 also show no effect on *P. infestans* colonisation compared to a GFP-EV control (Welch's t-test). Infection represented in box plots is measured as lesion diameter (mm) on inoculated leaves. Red dot indicates mean and blue dots are individual datapoints. The data represented in (a) and (b) are combinations of three independent experiments, consisting of at least 40 biological replicates each. Error bars indicate +/- SE. **(c)** Immunoblot shows that GFP-StNPH3 and GFP-StRPT2 proteins are stable when transiently expressed in *N. benthamiana*.

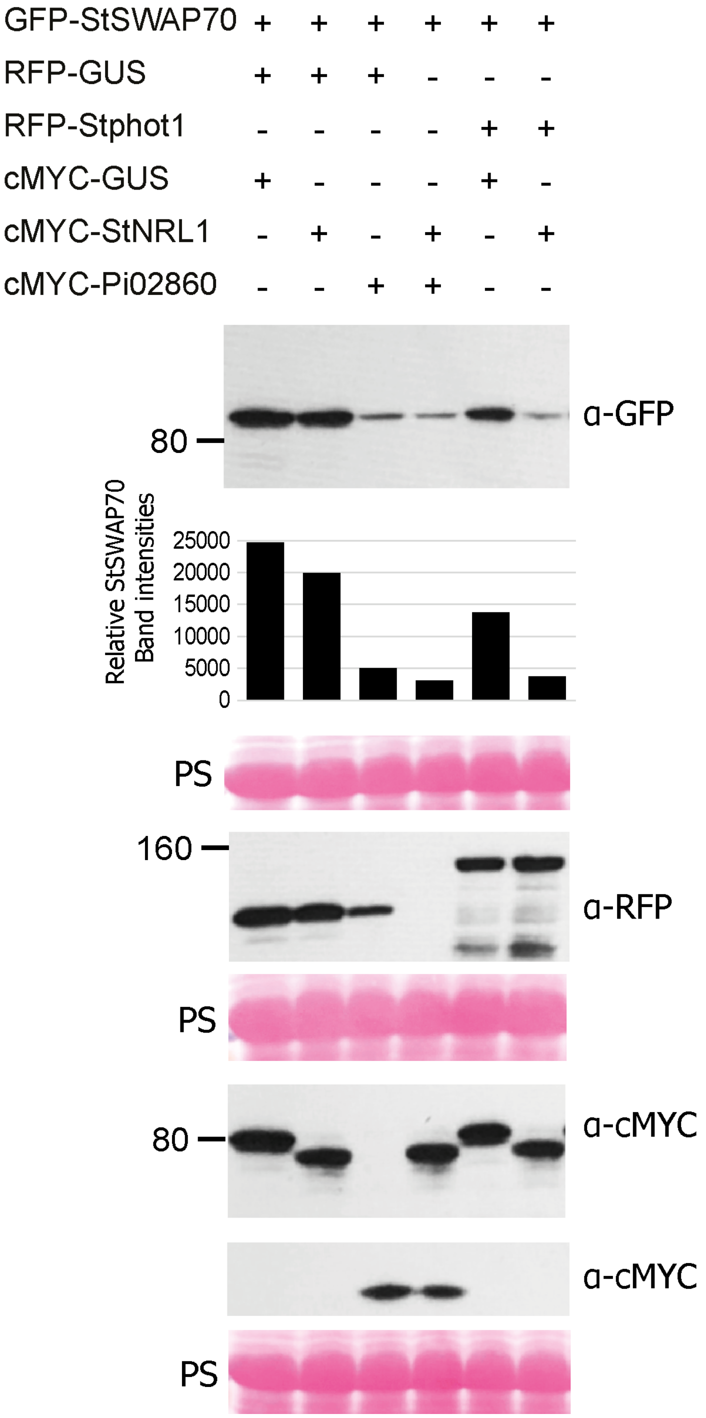

Fig. S3 Similar to StNRL1 and Pi02860, Stphot1 co-expression also reduces the abundance of StSWAP70.

Immunoblot shows decreased protein abundance of GFP-StSWAP70 when co-expressed with cMYC-StNRL1 and cMYC-Pi02860, individually or in combination, compared to cMYC-GUS control, as previously demonstrated (He & Naqvi et al., 2019). GFP-StSWAP70 stability is also reduced when co-expressed with RFP-Stphot1 and was further reduced when co-expressed with a combination of RFP-Stphot1 and cMYC-StNRL1.

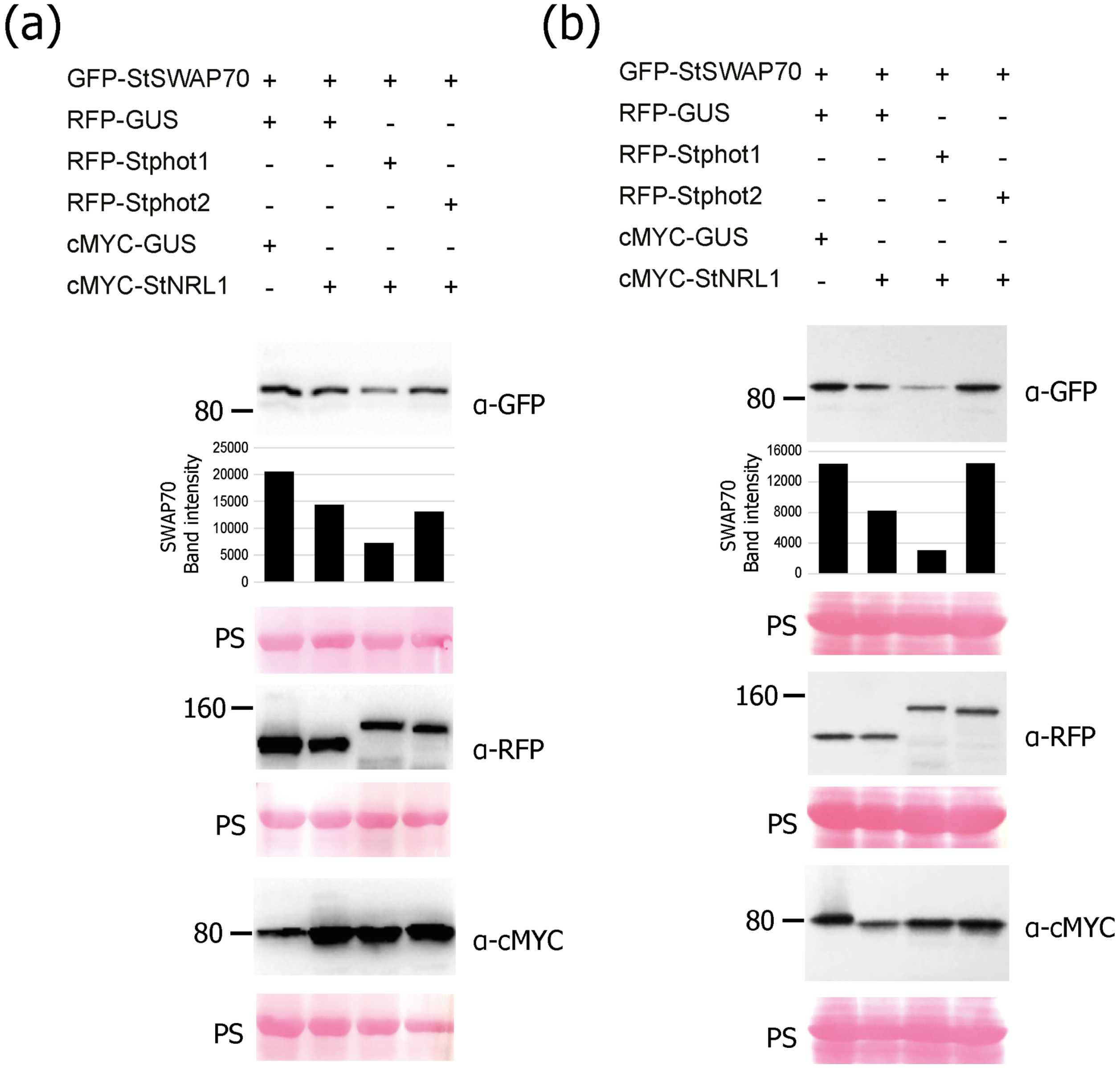

Fig. S4 Replicate Immunoblots of Fig. 3a.

(a) (b) Two replicate immunoblots show that, in the presence of cMYC-StNRL1, reduced GFP-

StSWAP70 abundance is stimulated specifically by co-expression with RFP-Stphot1 and not RFP-Stphot2 or the RFP-GUS control. Constructs expressed in *N. benthamiana* leaves are indicated by +. Protein sizes are represented in kilodaltons (kDa) and protein loading is shown by Ponceau staining (PS). The graphs below GFP-StSWAP70 show relative intensity of bands.

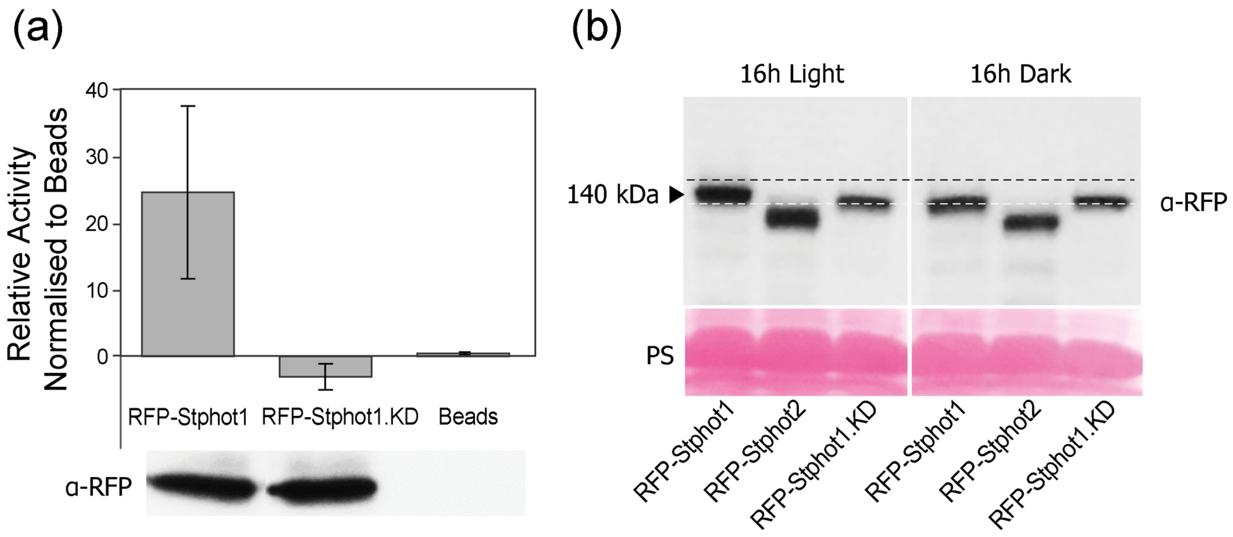

Fig. S5 Stphot1 mutant Stphot1.D832N is kinase-dead (Stphot1.KD).

(a) Graph showing kinase activity of RFP-p1 vs RFP-phot1.KD using beads as a control. Incubation of wildtype Stphot1 causes successful conversion of added ATP to ADP, while no ADP was detected in samples incubated with the kinases-dead form Stphot1.D832N. Graph showing ADP formation normalised to beads that were incubated with a protein extract from uninfiltrated leaves. Immunoblot below indicates equivalent loading of wild type and D832N mutant of Stphot1. Error bars indicate +/- SE. **(b)** Immunoblot showing mobility shifts of Stphot1 variants and Stphot2 in 16 h light conditions vs 16 h dark treatment. Protein samples were run on a 6.5 % acrylamide gel for optimised separation. The wildtype RFP-Stphot1 and Stphot2 show autophosphorylation and hence the mobility shift of WT proteins in light but not in the dark. The kinase-dead mutant RFP-Stphot1.KD does not show this shift in the light condition vs dark treatment indicating a lack of autophosphorylation. The mobility shift is indicated by black dotted line for Stphot1 and white for Stphot2.

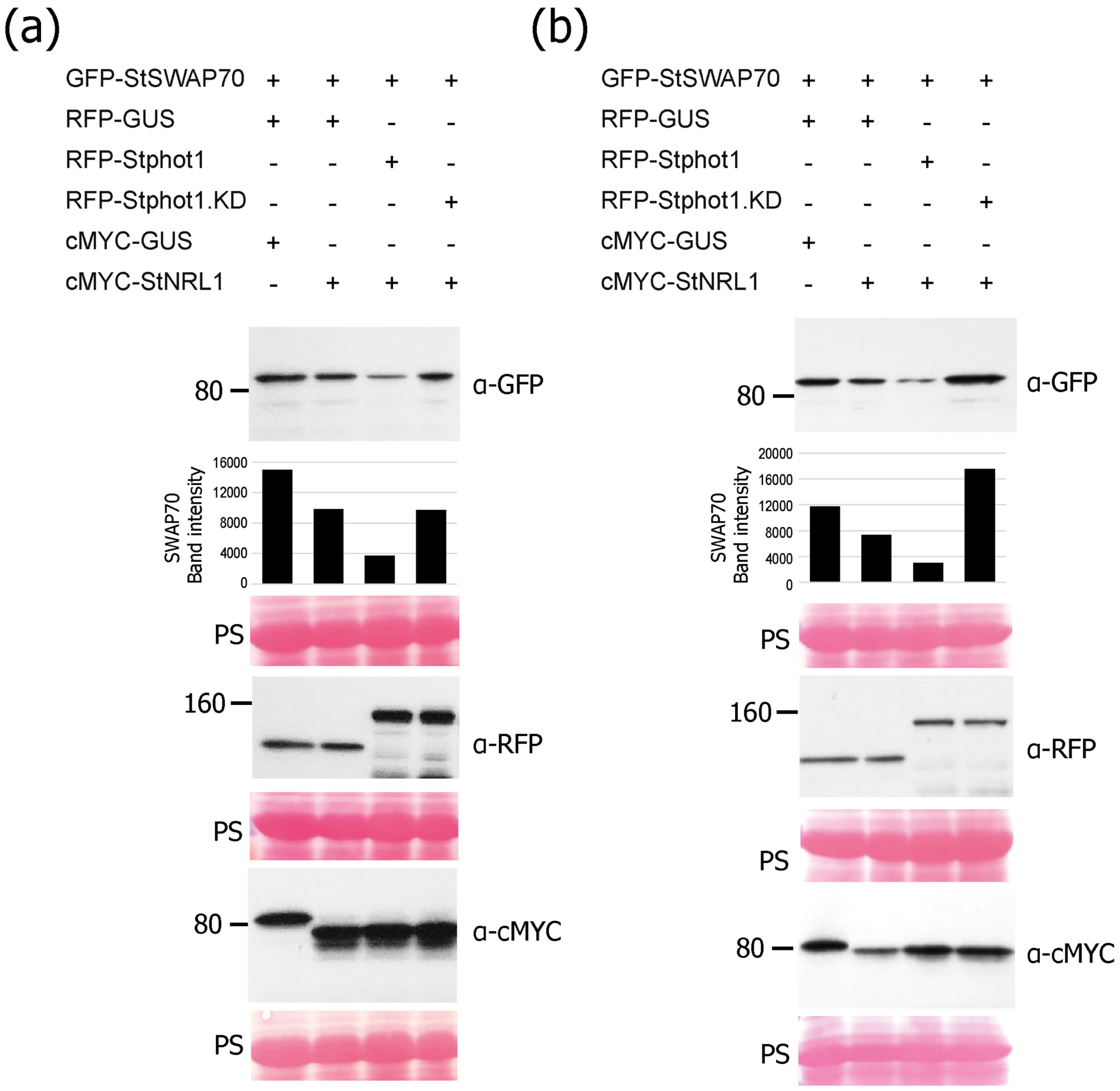

Fig. S6 Replicate Immunoblots of Fig. 5a*.*

(a) (b) Replicate immunoblots showing that the GFP-StSWAP70 protein level is reduced when co-expressed with either RFP-Stphot1+cMYC-GUS or RFP-Stphot1+cMYC-StNRL1 but not with the kinase-dead mutant RFP-Stphot1.KD and cMYC-StNRL1. Constructs expressed in *N. benthamiana* leaves are indicated by a plus sign (+). Protein sizes are represented in kilodaltons (kDa) and protein loading is shown by Ponceau staining (PS). The graph below shows the intensity of bands analysed

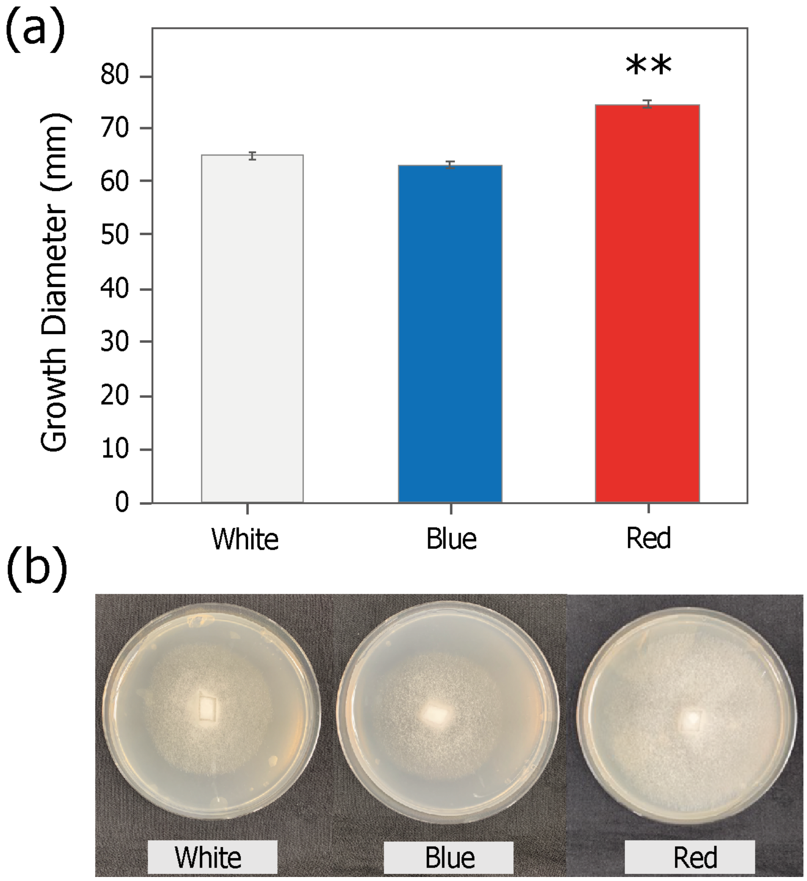

Fig. S7 *In vitro* growth of *P. infestans* under various light conditions*.*

P. infestans growth diameter under blue light is similar to under white LEDs. However, red light resulted in a faster growth when measured at day 10 (one-way ANOVA with Tukey's HSD Post-Hoc test, **p<0.001; n=20). Error bars indicate +/- SE. **(b)** Visual comparison of *P. infestans* growth diameters on rye agar plates, under white, red or blue light as indicated.

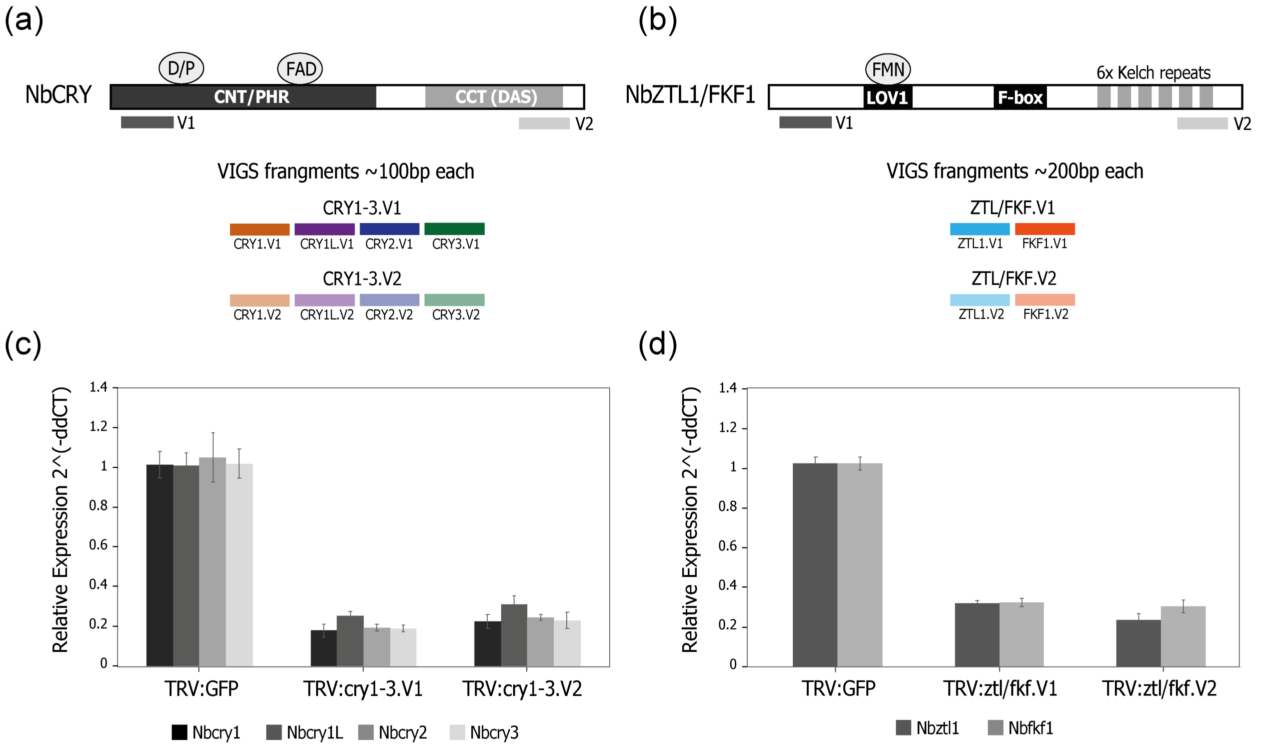

Fig. S8 Silencing of *CRYs, ZTL* and *FKF* genes in *N. benthamiana.*

(a), **(b)** Schematic diagrams for the *Nbcrys*, *Nbztl1/Nbfkf1* showing the areas used to generate the VIGS silencing constructs. Four constructs each for cry1, cry1L, cry2 and cry3 (a) and two for each ztl1 and fkf1 (b) were combined to achieve quadruple and double silencing constructs, respectively, at 5' and 3' ends of the coding sequence. **(c)**, **(d)** The graphs showing qRT-PCR analysis on leaves from silenced *N. benthamiana* plants indicates an 80 % reduction in all *Nbcrys* transcript levels in TRV:Nbcry1-3.V1 and V2 (c) and a 70 % reduction in *Nbztl1* and *Nbfkf1* transcripts in TRV:Nbztl/fkf.V1 and V2 constructs (d). TRV:GFP used as a control and EF1a was used to normalise the gene expression. The analysis was performed using the 2ddCt method and the error bars indicate SE. The data represented is a combination of three independent experiments.

Table S1 List of primers used in this study.

Gene	Task	Forward Primer	Reverse Primer
Stphot1<br>*Stphot2*<br>*StNPH3*<br>*StRPT2*	Cloning into pDONR from PUC57 vector	**Attb-1:** GGGGACAAGTTTGTACAAAAAAGCAGGC	**Attb-2:** GGGGACCACTTTGTACAAGAAAGCTGGGT
Stphot1	Stphot1 Kinase dead mutant	**Stphot1 D832N.F:**<br>GGGCATGTATCTTTAACGAATTTTGATCTTTCCTGTTTGAC	**Stphot1 D832N.R:**<br>GTCAAACAGGAAAGATCAAAATTCGTTAAAGATACATGCCC
Nbphot1	Nbphot1 VIGS Construct	**Nbphot1_V1.F:**<br>ATCCGAATTCCAATCTCCTCCTCTTATACCACCA<br>**Nbphot1_V2.F:**<br>ATCCGAATTCGCTCTTGGCATTCTTTTATACG	**Nbphot1_V1.R:**<br>GGTAGTTAACGTCTGAGTAATCGGAGAAGACAAT<br>**Nbphot1_V2.R:**<br>GGTAGTTAACGGGATTACAAGACACCCTTGAG
Nbphot2	Nbphot1 VIGS Construct	**Nbphot2_V1.F:**<br>ATCCGAATTCAGGTGGAAAGTCGGCTAGTAGT<br>**Nbphot2_V2.F:**<br>ATCCGAATTCATGAGATGCTCTATGGGCGAA	**Nbphot2_V1.R:**<br>GGTAGTTAACCTCCAATGAATTCTTGCCTCTT<br>**Nbphot2_V2.R:**<br>GGTAGTTAACAGTCAATATCCTTGGTGCCT
Nphot1/2	Nbphot1/2 Double VIGS Construct	**Nbphot_double1.F:**<br>ATTGTCTTCTCCGATTACTCAGACAGGTGGAAAGTCGGCTAGTAGT<br>**Nbphot_double2.F:**<br>CTCAAGGGTGTCTTGTAATCCCATGAGATGCTCTATGGGCGAA	**Nbphot_double1.R:**<br>ACTACTAGCCGACTTTCCACCTGTCTGAGTAATCGGAGAAGACAAT<br>**Nbphot_double2.R:**<br>TTCGCCCATAGAGCATCTCATGGGATTACAAGACACCCTTGAG
NbEf1a	qRT-PCR	**qPCR.NbEf1a-F:** TGGACACAGGGACTTCATCA	**qPCR.NbEf1a-R:**CAAGGGTGAAAGCAAGCAAT
Nbphot1	qRT-PCR	**Nbphot1.qPCR-F2:** AGATGCCTTATCAACATT	**Nbphot1.qPCR-R2:**CCAATTCCTGCCTATAAC
Nbphot2	qRT-PCR	**Nbphot2.qPCR-F1:** GAAGGCTCTTGAACTACA	**Nbphot2.qPCR-R1:** GCATTCCTATAAACTTGATTG
Nbcry1	qRT-PCR	**qPCR.NbCRY1-F:** GGACCGTTAATTGAGTATTC	**qPCR.NbCRY1-R:**CAGAGAACTTGCTTGATAC
Nbcry1L	qRT-PCR	**qPCR.NbCRY1L-F:**AATCTACTGGTGCTACTCA	**qPCR.NbCRY1L-R:**CCTTCTTCATCATTAACTTCC
Nbcry2	qRT-PCR	**qPCR.NbCRY2-F:**GTTCGTGACCACAATATC	**qPCR.NbCRY2R:**CCATCATCATCGTAGACTT
Nbcry3	qRT-PCR	**qPCR.NbCRY3-F:**AATAGAGGACGACAGATTG	**qPCR.NbCRY3-R:**TGCCATAGTTGGAGCAAGGA
Nbztl1	qRT-PCR	**qPCR.NbZTL1-F2:**TCCAACAGAAGAGAAGCCTACA	**qPCR.NbZTL1-R2:**ATACATGTGCTATGTCCCCAAG
Nbfkf1	qRT-PCR	**qPCR.NbFKF1-F1:**ATTCCTTCTTCTCAGCAGCCAAT	**qPCR.NbFKF1-R1:**GAAACAACGATAGAAGACGGCG

Table S2 Sequence of gene fragments used in antisense orientation for virus-induced gene silencing constructs in *N. benthamiana***.**

Gene Names	Accession numbers	VIGS Fragments
Nbphot1.V1	NbS00003596g0009	CAATCTCCTCCTCTTATACCACCACTCCCCCGAGATCCAAGAGGCTCACTAGAAGTATTCAACCCATCAACTTACTCTATTTCCCGGCCAAAAAATCCAGTTTTCCGATT<br>ATCACAGCCGTCGTGGAACAATAATTGGGCCGAGCCCGAGCTTGAGCCCATTAAAAGAAGCAGCAGCATCCCTGAAACAGAAGAAGAATCAGAGCCTATAGTATCCAATA<br>ATAATGATATTAAAGAGGAAACTATTGCCACGTCATGGATGGCAATCAAAGACCCAATTATTATTACTCCAATTTCAACGTCGCAATTGTCTTCTCCGATTACTCAGAC
Nbphot1.V2		GCTCTTGGCATTCTTTTATACGAAATGCTGTATGGTTACACACCATTCAGGGGAAAGACAAGGCAAAAGACATTCTCAAATATATTACACAAGGATTTAAAATTCTCAGG<br>AAGCATACAGGTTAGTCTCCAAGGAAAGCAGTTCATGTATAGATTGTTGCATAGAGATCCCAAAAACAGGTTAGGATCCCGCGAAGGAGCAAATGAAATCAAGCAGCATC<br>CATTCTTTCGAGGCGTGAATTGGGCATTAGTTCGATGCATGAACCCTCCAAAACTAGATGCTCCTCAAGGGTGTCTTGTAATCCC
Nbphot2.V1	NbS00055894g0005	AGGTGGAAAGTCGGCTAGTAGTAGAAATGAGGGTATTGATGTGCAAGAAGTTCCAATGAAAGGGGTGGAATCTGGGACTATGACAGGAACAAATAAGCCAATGAACAAGT<br>GGATGGCCTTTGACCCGAATGGGAAAAAAGGAGAAGATAACGGCAATGCAAATAGTCAGATCCCTAGTGAACCCAGCATAGCAGCGAGGGCTGCAGAGTGGGGATTGACA<br>GTGAGGACAGATGTTGGAGAAGGTAGCTTTCACGCAATTAGCAGAAGCGGAGAGAATTCATTTGCAGATGGGGAAAGAGGCAAGAATTCATTGGAG
Nbphot2.V2		ATGAGATGCTCTATGGGCGAACGCCATTTAGAGGAAAGAATAGGCAGAAGACATTTGCCAACATCCTAAACAAGGACCTCACCTTCCCCAGCAGCATTCCGGTATGCCTT<br>GCAGCTAGACAGTTGATTCATGCTTTGCTAAATAGAGATCCAGCCAGCCGTTTAGGATCAAATGGCGGTGCAAGTGAGATCAAAGATCATCCTTTCTTCCGTGGAATAAA<br>TTGGCCACTAATTCGCTGCATGACTCCACCACCACTAGACGCACCCCTTCAGTTAATTGGAAAACAATCAGGCACCAAGGATATTGACT
Nbphot1/2.V1	NbS00003596g0009<br>NbS00055894g0005	CAATCTCCTCCTCTTATACCACCACTCCCCCGAGATCCAAGAGGCTCACTAGAAGTATTCAACCCATCAACTTACTCTATTTCCCGGCCAAAAAATCCAGTTTTCCGATT<br>ATCACAGCCGTCGTGGAACAATAATTGGGCCGAGCCCGAGCTTGAGCCCATTAAAAGAAGCAGCAGCATCCCTGAAACAGAAGAAGAATCAGAGCCTATAGTATCCAATA<br>ATAATGATATTAAAGAGGAAACTATTGCCACGTCATGGATGGCAATCAAAGACCCAATTATTATTACTCCAATTTCAACGTCGCAATTGTCTTCTCCGATTACTCAGACA<br>GGTGGAAAGTCGGCTAGTAGTAGAAATGAGGGTATTGATGTGCAAGAAGTTCCAATGAAAGGGGTGGAATCTGGGACTATGACAGGAACAAATAAGCCAATGAACAAGTG<br>GATGGCCTTTGACCCGAATGGGAAAAAAGGAGAAGATAACGGCAATGCAAATAGTCAGATCCCTAGTGAACCCAGCATAGCAGCGAGGGCTGCAGAGTGGGGATTGACAG<br>TGAGGACAGATGTTGGAGAAGGTAGCTTTCACGCAATTAGCAGAAGCGGAGAGAATTCATTTGCAGATGGGGAAAGAGGCAAGAATTCATTGGAG
Nbphot1/2.V2		GCTCTTGGCATTCTTTTATACGAAATGCTGTATGGTTACACACCATTCAGGGGAAAGACAAGGCAAAAGACATTCTCAAATATATTACACAAGGATTTAAAATTCTCAGG<br>AAGCATACAGGTTAGTCTCCAAGGAAAGCAGTTCATGTATAGATTGTTGCATAGAGATCCCAAAAACAGGTTAGGATCCCGCGAAGGAGCAAATGAAATCAAGCAGCATC<br>CATTCTTTCGAGGCGTGAATTGGGCATTAGTTCGATGCATGAACCCTCCAAAACTAGATGCTCCTCAAGGGTGTCTTGTAATCCCATGAGATGCTCTATGGGCGAACGCC<br>ATTTAGAGGAAAGAATAGGCAGAAGACATTTGCCAACATCCTAAACAAGGACCTCACCTTCCCCAGCAGCATTCCGGTATGCCTTGCAGCTAGACAGTTGATTCATGCTT<br>TGCTAAATAGAGATCCAGCCAGCCGTTTAGGATCAAATGGCGGTGCAAGTGAGATCAAAGATCATCCTTTCTTCCGTGGAATAAATTGGCCACTAATTCGCTGCATGACT<br>CCACCACCACTAGACGCACCCCTTCAGTTAATTGGAAAACAATCAGGCACCAAGGATATTGACT
Nbcry1-3.V1	**NbCry1**<br>Niben101Scf02751g04010/Niben101Scf01980g0900<br>**NbCry1-Like**<br>Niben101Scf01167g05010/Niben101Scf02537g1500<br>**NbCry2**<br>Niben101Scf06017g01010/Niben101Scf06779g00008<br>**NbCry3**<br>Niben101Scf00360g00006	AGTGGTGGTTGTAGCATAGTATGGTTTAGGAGGGATCTGAGGTTAGAAGATAACCCAGCACTGGCTGCTGGAGTGAGAGCAGGAGCAGTGATTGCATTGTGTCAGGTGGT<br>GGTTGTAGCATAGTATGGTTTAGAAGGGATCTGAGAGTAGAAGATAATCCAGCTTTAGCTGCAGGAGTAAGAGCAGGAGCAGTAATTGGAGAGCAATTGTAAGACAATTG<br>TGTGGTTTAGGAGGGATCTGAGAATTGAGGATAATCCAGCTTTAGCAGCAGCTGCTAGGAATGGGAGTGTATTTCCAGTGTTTGAATCTGAACGTGATGATCAAACAACC<br>TCTTTCTTTAACGAAACTCACTACATCTTCTTTAAGCCAATTAAATCACACCCTTTTCAAATTCTCCGGAAG
Nbcry1-3.V2		AGACTGCCCACTGAATGGATACACCACCCTTGGGATGCACCAGAACCTGTACTTCAAGCTGCTGGTATTGAGCTTGGTTCCAATTATCCTCTTCCAATTGTTGAGCCGTC<br>ACACAACGAGCTCGAACAAACGCCACTCCGCCGCAGTTTAATTTTGTGGTTGGTAGGAGAAATTCTGAAGATTCTACAGCAGAATCTTCCAGTAGTACCCAAAGGTTGTA<br>AAGGGAAAGGTGCCTTTCCCGAGCAGTTCATCTTATGATCAGAGGGTGCCATCAATGCAAAAGGGGAGCACAAATAAGAAGAGACCAAAGCCAGTCGAAGAAGAGAGCTG<br>AATGGTTTGAGACATGCCTCTTAGATTATGATCCTTGCTCCAACTATGGCAACTGGACATATGGAGCAGGTGTTGGCAATGACCCTAGAGAAGA
Nbztl1	NbS00009879g0004/<br>NbS00038815g0006	ATGAAGCGATAACTCACATTATTGGCATTCAGTTCTTCAAGGAAGTAAATATTGATTTGGGTCCACTTCCAGGATCCTTGGTAAAGGAGCCTACAAGACTACTGGATAAG<br>TACCGTTCTAGCCTATCTTCTAGTGGACCAGTCTCAGAAGGAAACCGCACTATCAGTCGTGGGTTTTGTGGTATATTGCAGTTGAGTGATGAAGTCCTAGCCCTCAAGAT<br>TCTTTCACGGTTGACCGCATGGGGAGCAGATGTCACAGGGGTGCTGGAACACATGACTAAAAGATTAGCTTGGGGGCGCCTGGCTAGGGAGCTAACCACTCTTGAAGCCG<br>TTTGTTGGAAGAAACTTACAGTTGGAGGGGCTGTAGAGCCTTCACGTTGCAATTTCAGTGCATGCGCTGCAGGGAACCGGCTGGTGTTATTTGGAGGGGAAGGTGTTAAT<br>ATGCAGCCAATGGACGATACA
Nbfkf1	NbS00025471g0004.1	GTCTTTGTGCTGGATTTGGATGCCAAACTACCAACTTGGCGTGAAATCTCTAGTTTGGCGCCTCCGCTTCCAAGATCTTGGCACAGCTCCTGTACCTTGGATGGTACCAA<br>GTTGATAGTCTCTGGTGGTTGTGCAGATTCTGGTGTGCTTCTAAGTGATACTTTCCTGCTTGATCTGTCTATAGAGAAGCCTGTGTGGCGTGAGATACCAGTGACGCGTC<br>AAGATTGGGACACTCACTCTCTGCTTATGGGAAGACAAAAATTCTAATGTTTGGTGGACTTGCCAAGAGTGGTCACTTGCGACTAAGATCAGGGGAATCATACACGATTG<br>ACTTGGAGGACGAAAGGCCACAGTGGAGGCAGCTTGAGTGTGGGGCGTTCACAGGAGGAGGAAGTCAGAATGCTGTGATTCCTCCTCCAAGACTTGATCACGTCGCTGTA<br>ACCATGCCTTG
